# Transgenerational inheritance of altered H3K27me3 in wild-type *Caenorhabditis elegans*

**DOI:** 10.1038/s44318-026-00907-9

**Published:** 2026-09-18

**Authors:** Isa Özdemir, François-Xavier Stubbe, Kamila Delaney, Joanna M Wenda, Florian A Steiner

**Affiliations:** Department of Molecular and Cellular Biology, Section of Biology, Faculty of Sciences, University of Geneva, Geneva, Switzerland

**Keywords:** Chromatin, Transcription & Genomics, Genetics, Gene Therapy & Genetic Disease

## Abstract

Epigenetic inheritance provides mutation-free mechanisms for phenotypic adaptation, including modifications to DNA or DNA-associated proteins that create patterns of heritable gene regulation. However, the limits and regulation of this inheritance remain incompletely understood. Here, we developed a *C. elegans* system to study the transgenerational epigenetic inheritance of H3K27me3, a conserved histone posttranslational modification associated with gene repression. We find that transiently induced alterations of the genome-wide H3K27me3 landscape and the associated fertility defects persist for many generations in genetically wildtype descendants under selective pressure. We uncover that the inheritance of the altered H3K27me3 landscape involves an initiation phase that relies on SET-32/H3K23me3 and a maintenance phase that requires MES-4/H3K36me3. Our results indicate that epigenetic inheritance can act as a mutation-independent, heritable mechanism of adaptation.

## Synopsis

Limits and regulation of epigenetic inheritance remain incompletely understood. This work shows that an altered genomic distribution of the histone modification H3K27me3 can be transmitted across many *C. elegans* generations through the successive activity of SET-32 and MES-4, which antagonize H3K27me3 deposition.

- Altered H3K27me3 landscapes can be inherited for at least 15 generations in *C. elegans*.
- Maintenance of the altered H3K27me3 landscape is metastable and can revert to wild-type in every individual and every generation.
- Epigenetic inheritance can be divided into an initiation phase that requires SET-32-mediated deposition of H3K23me3, and a maintenance phase that depends on MES-4-mediated deposition of H3K36me3.

## Abstract (web summary)

Altered genomic distribution of the histone modification H3K27me3 can be transmitted across many generations through the successive activity of SET-32 and MES-4, which antagonize its deposition.

## Graphical abstract

**Figure:**
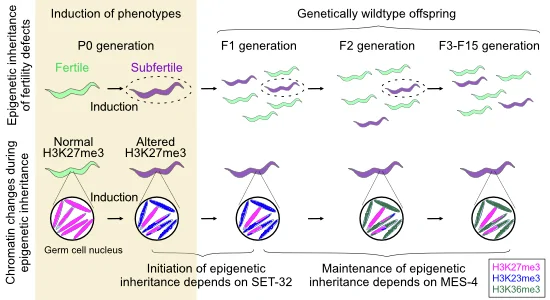

## Introduction

Natural selection is based on the survival and reproduction of individuals with the best-adapted traits for a given environment, so that the genomes of these individuals are more likely to be inherited by the next generations. Adaptation relies on the random occurrence of mutations in the genome and is therefore slow and untargeted. However, epigenetic factors such as small RNAs, DNA methylation, or histone posttranslational modifications (PTMs) can affect gene expression in a heritable way, and can change in response to environmental influences, providing a potential mechanism for fast, targeted, and reversible adaptation (Waddington, 1942).

Histone PTMs can act as chromatin-based epigenetic factors to influence gene expression without alterations in DNA sequence. Therefore, the mitotic transmission of histone PTMs is one of the most-studied subjects of epigenetic inheritance (EI) (Özdemir and Steiner, 2021). There is consensus for a semi-conservative chromatin replication model, in which the parental chromatin serves as a template to replicate chromatin landscapes through the action of reader and writer proteins that recognize and catalyze histone PTMs, respectively (Alabert and Groth, 2012). Thus, in addition to the genetic material, the landscape of some histone PTMs is replicated in *cis* during the cell cycle. Typical examples of such “replicative EI” are the maintenance of repressive histone PTMs on “selfish” elements (Yang et al, 2019), or the barrier to cellular reprogramming in differentiated cell lineages (Nicetto and Zaret, 2019). However, not all histone PTMs are heritable. In cases such as the deposition of histone PTMs in response to environmental stimuli, the epigenetic remodeling during the germline-to-soma transition, or the lineage differentiation during development, “reconstructive EI” takes place, where histone PTMs are targeted de novo to specific genomic regions (Özdemir and Steiner, 2021; Alabert and Groth, 2012; Stewart-Morgan et al, 2020; Reverón-Gómez et al, 2018; Petryk et al, 2021; Yu et al, 2018; Xia et al, 2019; Zheng et al, 2016). The epigenome of any given cell results from both replicative EI and reconstructive EI, thereby enabling the germ line to give rise to highly specialized, yet totipotent germ cells (Jablonka, 2013, 2017; Margueron and Reinberg, 2010).

Examining EI from parents to offspring or across multiple generations is more challenging due to the scarcity of a practical experimental model. Long generation times, epigenome mixing during sexual reproduction, and varying epigenetic buffering capacities among individuals pose challenges for studying transgenerational epigenetic inheritance (TEI) in mammalian systems. In contrast, self-fertilizing, isogenic *Caenorhabditis elegans* is convenient for such studies owing to the fast reproduction, invariant development, and large brood sizes with little genetic variability between individuals, while at the same time sharing similar chromatin-based epigenetic systems with humans. *C. elegans* has been extensively used as a model system for the study of small RNA-based TEI, which in many cases involves changes to the histone PTMs at the targeted genomic loci (Woodhouse et al, 2018; Kalinava et al, 2018; Buckley et al, 2013). However, whether TEI can be based directly on initial chromatin changes is less well understood (Fitz-James and Cavalli, 2022).

Typically, investigating EI of histone PTMs relies on the genetic removal of reader, writer or eraser proteins, and the observation of the subsequent changes in the patterns of the histone PTMs (defects in replicative EI), or the failure to initiate them de novo (defects in reconstructive EI). For instance, the Polycomb histone PTM H3K27me3 is essential for germ line maintenance and cell differentiation (Cockrum and Strome, 2022; Gaydos et al, 2014, 2012; Kaneshiro et al, 2022, 2019). H3K27me3 is associated with repressed genes (Cao et al, 2002) and can be subjected to replicative EI and reconstructive EI. Mutating Polycomb group genes (PCGs) results in progressive and detrimental phenotypes such as mortal germ line (Mrt) or maternal-effect sterility (Mes) in *C. elegans*, which are linked to the failure to maintain or de novo initiate H3K27me3-mediated gene silencing. While such analyses allow mechanistic insight into the short-term maintenance of H3K27me3, the sterility phenotypes limit the study of H3K27me3 long-term inheritance (Gaydos et al, 2012, 2014).

To overcome these challenges and study the long-term inheritance of H3K27me3 patterns across generations, we developed a *C. elegans* model to alter H3K27me3 landscapes by the transient expression of a mutant histone H3.3 (H3.3K27M). We then analyzed the inheritance of the altered H3K27me3 landscapes and the associated fertility defects among the wildtype offspring of the subsequent generations. H3.3K27M was originally identified as a dominant-negative driver mutation of human high-grade gliomas, and we previously showed that its expression in *C. elegans* leads to an autosomal loss of H3K27me3 in germ cells, resulting in aberrant gene expression, endomitotic oocytes, and low fertility (Delaney et al, 2019; Lewis et al, 2013). Here, we show that the H3K27me3 landscapes altered by H3.3K27M expression and the associated fertility defects are epigenetically inherited for at least 15 generations of genetically wildtype offspring in a metastable way. We found that the inheritance is divisible into an initiation phase relying on SET-32-mediated deposition of H3K23me3, and a maintenance phase requiring MES-4-mediated deposition of H3K36me3. Our findings provide a mechanistic understanding of how organisms can transiently adapt their epigenomes and inherit these changes in subsequent generations.

## Results

### An inducible *C. elegans* system to transiently alter H3K27me3 epialleles

**Figure 1:**
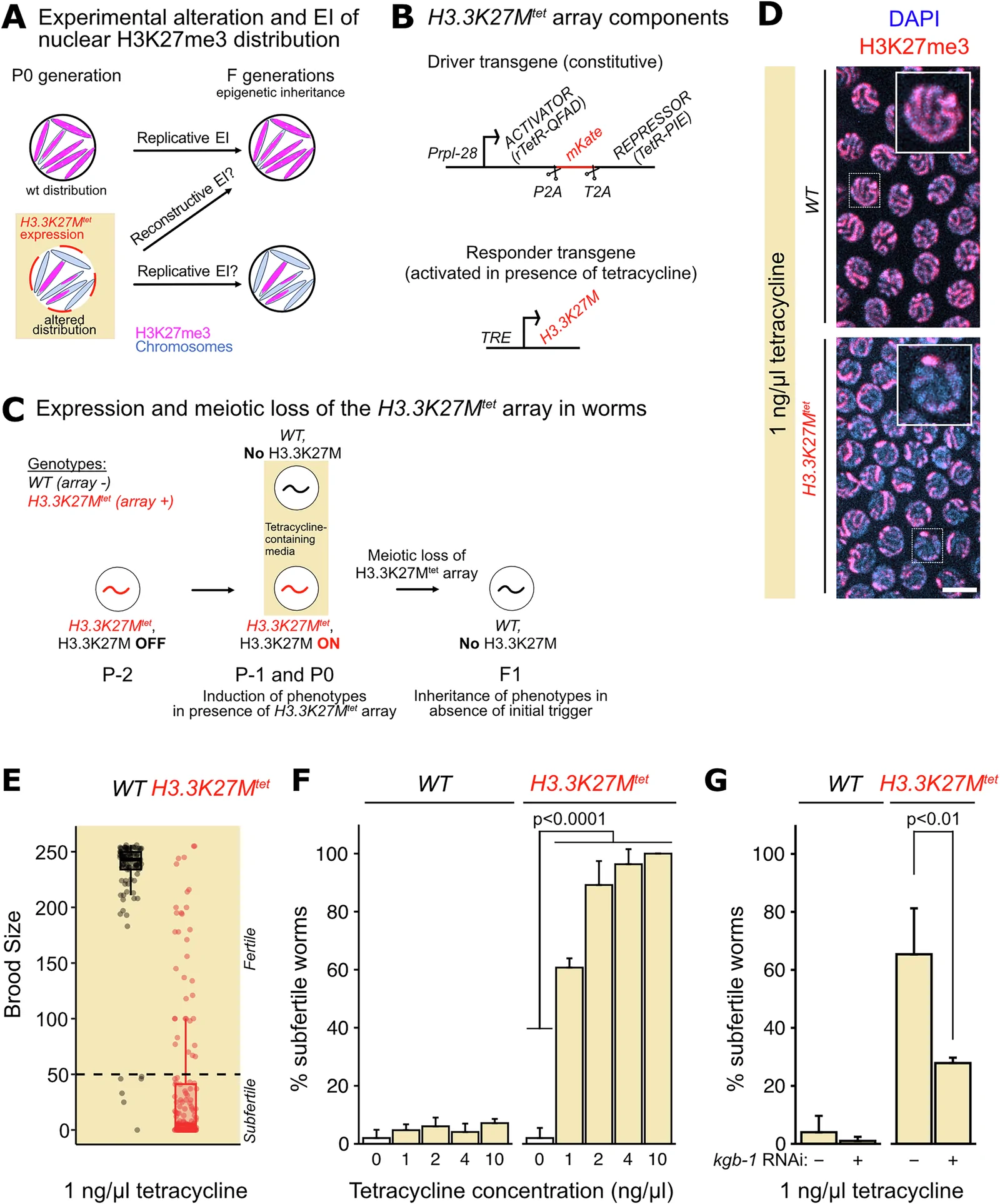
An inducible *C. elegans* system to transiently alter H3K27me3 epialleles. (**A**) Schematic representation of the distribution of H3K27me3 in pachytene nuclei investigated in this study. In wildtype individuals, H3K27me3 (magenta) is distributed on all chromosomes (blue). The beige background signifies the experimental induction of H3.3K27M expression. Upon induced expression of H3.3K27M (nucleus with red dashed outline), H3K27me3 is restricted to the X chromosome and a few autosomal loci. The study investigates if this altered distribution is epigenetically inherited to the F generations that no longer express H3.3K27M (replicative EI), or if the wildtype distribution is recovered in the F generations (reconstructive EI). (**B**) Schematic representation of the tetracycline-inducible *H3.3K27M*^*tet*^ transgenic array. The array consists of two co-injected transgenes: the driver transgene (top) is constitutively co-expressing the activator (rtTA(Q); rTetR fused to the QFAD activation domain from the fungal transcription factor QF), mKate (fluorescent marker), and the repressor (tTS; TetR fused to the repressor domain from *C. elegans* PIE-1), and the responder transgene (bottom) consists of a regulatory tetracycline response element (TRE; *7xTetO::Ppes-10*) controlling the coding sequence for OLLAS-tagged H3.3K27M. ACTIVATOR, mKate and REPRESSOR are separated by self-cleaving P2A and T2A peptides. In the absence of tetracycline, the repressor binds to the TRE to prevent expression of the responder transgene, and upon exposure to tetracycline, the ACTIVATOR replaces the REPRESSOR at the TRE and drives the expression of H3.3K27M. For details, see methods and (Mao et al, 2019). (**C**) Schematic representation of the activation and meiotic loss of the *H3.3K27M*^*tet*^ extrachromosomal array. Worms containing the *H3.3K27M*^*tet*^ array are recognizable by their constitutive red fluorescence and do not express H3.3K27M unless they are grown on tetracycline-containing media (beige background). The extrachromosomal array is meiotically unstable, and worms carrying the array produce genetically wildtype offspring (WT, shown in black) that do not express H3.3K27M. This system can therefore be used to study whether the phenotypes induced by the expression of H3.3K27M in the P0 generation are inherited by genetically wildtype offspring in the F generations. (**D**) Representative images from immunostaining of H3K27me3 in wildtype (WT) and *H3.3K27M*^*tet*^ pachytene nuclei counterstained with DAPI (blue) from animals grown on media containing 1 ng/µl tetracycline. Scale bar represents 5 µm. (**E**) Brood size distribution of wildtype (WT, black) and *H3.3K27M*^*tet*^ (red) animals grown on media containing 1 ng/µl tetracycline. Each dot represents the complete brood size of an individual worm (*n* = 102 for WT and *n* = 156 for *H3.3K27M*^*tet*^). Box and whiskers plots show medians, first and third quartiles (box) and 10th and 90th percentiles (whiskers). The dashed line demarks the arbitrary threshold between the *fertile* and *subfertile* phenotypes. (**F**,** G**) Percentages of subfertile wildtype (WT) and *H3.3K27M*^*tet*^ animals (20 < *n* < 61 animals per condition). Means and standard errors are shown. *P* values are from two-way ANOVA with Sidak’s multiple comparison tests (*p* = 3.0e^−8^ in (**F**) and *p* = 0.008) in (**G**). (**F**) Worms were grown on media containing 0, 1, 2, 4, or 10 ng/µl tetracycline. (**G**) Worms were grown on media containing 1 ng/µl tetracycline and treated with *control RNAi* or *kgb-1(RNAi)*.

To investigate the inheritance of H3K27me3 patterns across generations, we first designed an approach to transiently alter the H3K27me3 landscape in the *C. elegans* germ line. We aimed to induce changes in the parental generation to mimic an environmental event, and then to monitor the inheritance (replicative EI) or recovery (reconstructive EI) in the offspring generations in the absence of the initial trigger (Fig. 1A). We had previously shown that the expression of a dominant-negative lysine 27 to methionine mutant of histone H3.3 (H3.3K27M) results in a redistribution of H3K27me3 in *C. elegans* germ cells, where it becomes restricted to the X chromosomes and a few autosomal domains (Delaney et al, 2019). As a consequence of this redistribution, genes in the autosomal regions that lose H3K27me3 become ectopically expressed, resulting in endomitotic oocytes and low fertility (Delaney et al, 2019). The genome-wide redistribution of H3K27me3 is easily visible by immunofluorescence (IF), enabling an assessment of cell-to-cell variability within a single worm. This provides a major advantage over TEI studies using reporter genes, where the detection of chromatin changes requires ChIP-seq experiments, and the signal from many cells and individuals is averaged. The previously demonstrated causal relationship between the H3.3K27M-induced chromatin changes and reduced fertility provided us with a phenotypic readout on the level of individual worms (Delaney et al, 2019).

To restrict the expression of the H3.3K27M protein to the parental generation, we designed a tetracycline-inducible transgene (*H3.3K27M*^*tet*^) (Fig. 1B). The H3.3K27M responder transgene is under the control of a constitutive driver transgene that expresses both an activator (rtTA(Q); rTetR fused to the QFAD activation domain from the fungal transcription factor QF) and a repressor (tTS; TetR fused to the repressor domain from *C. elegans* PIE-1) (Mao et al, 2019). In the absence of tetracycline, the repressor binds to the Tetracycline Response Element (TRE) and prevents expression of the responder transgene, and in the presence of tetracycline, the activator replaces the repressor at the TRE to drive H3.3K27M expression (indicated by a beige background or labeling in all the figures) (Fig. 1B, C). The inducible expression of H3.3K27M mimics an environmental event that changes the H3K27me3 epigenome, and we aimed to investigate the transmission of the changed epigenomes to the wild-type offspring. To exclude the analysis of worms with leaky expression of the transgene in the inheriting generations, we delivered the transgenes in the form of an extrachromosomal array that has a low meiotic transmission rate and is lost in part of the offspring. The driver transgene also encodes a fluorescent mKate protein, so that worms containing the array (*H3.3K27M*^tet^) can easily be distinguished by their red fluorescence from worms that have lost the array (WT). This allowed us to study the inheritance of altered H3K27me3 landscapes in genetically wildtype offspring (Fig. 1C).

First, we verified by IF that *H3.3K27M*^tet^, but not wildtype worms, showed a redistribution of the H3K27me3 landscape upon tetracycline treatment, with a global depletion of the H3K27me3 signal on autosomes, and at least a partial retention on chromosome X (Fig. 1D). The persistence of the H3K27me3 signal on the X chromosome upon *H3.3K27M*^*tet*^ induction was previously verified and explained by both low levels of H3.3 and high pre-existing levels of H3K27me3 on the X chromosome (Delaney et al, 2019). The *H3.3K27M*^*tet*^-induced changes in the H3K27me3 landscape led to a strong decrease in brood sizes at 25 °C in most of the worms, with some escapers (Fig. 1E). To use fertility as a selection criterion, we classified the phenotypes into subfertile, arbitrarily defined as 0–50 embryos (including animals without germ line), and fertile, defined as >50 embryos. Wildtype worms typically had 200–250 offspring at 25 °C (Fig. 1E). The subfertile phenotype was detected almost exclusively in worms carrying the *H3.3K27M*^tet^ array, and only upon tetracycline induction (Fig. 1E,F). It became more penetrant with increasing tetracycline concentrations used for *H3.3K27M*^tet^-induction (Fig. 1F). We selected a level of induction that caused the fertility defects, but also allowed for sufficient fertility to propagate the next generations (1 ng/µl tetracycline). Consistent with our previous observations, the *H3.3K27M*^tet^-induced fertility defects were partially suppressed by the depletion of *kgb-1*, a MAPK subfamily serine-threonine kinase that becomes derepressed in the germline of worms expressing H3.3K27M (Fig. 1G) (Delaney et al, 2019). Taken together, the system allowed us to use tetracycline treatment of worms carrying the *H3.3K27M*^tet^ array to induce a redistribution of H3K27me3 that resulted in the expected and previously characterized fertility defects, and then to select genetically wildtype offspring that had lost the array for further analysis.

### Altered H3K27me3 landscapes and the resulting fertility defects are epigenetically inherited for at least 15 generations in genetically wildtype progeny

**Figure 2:**
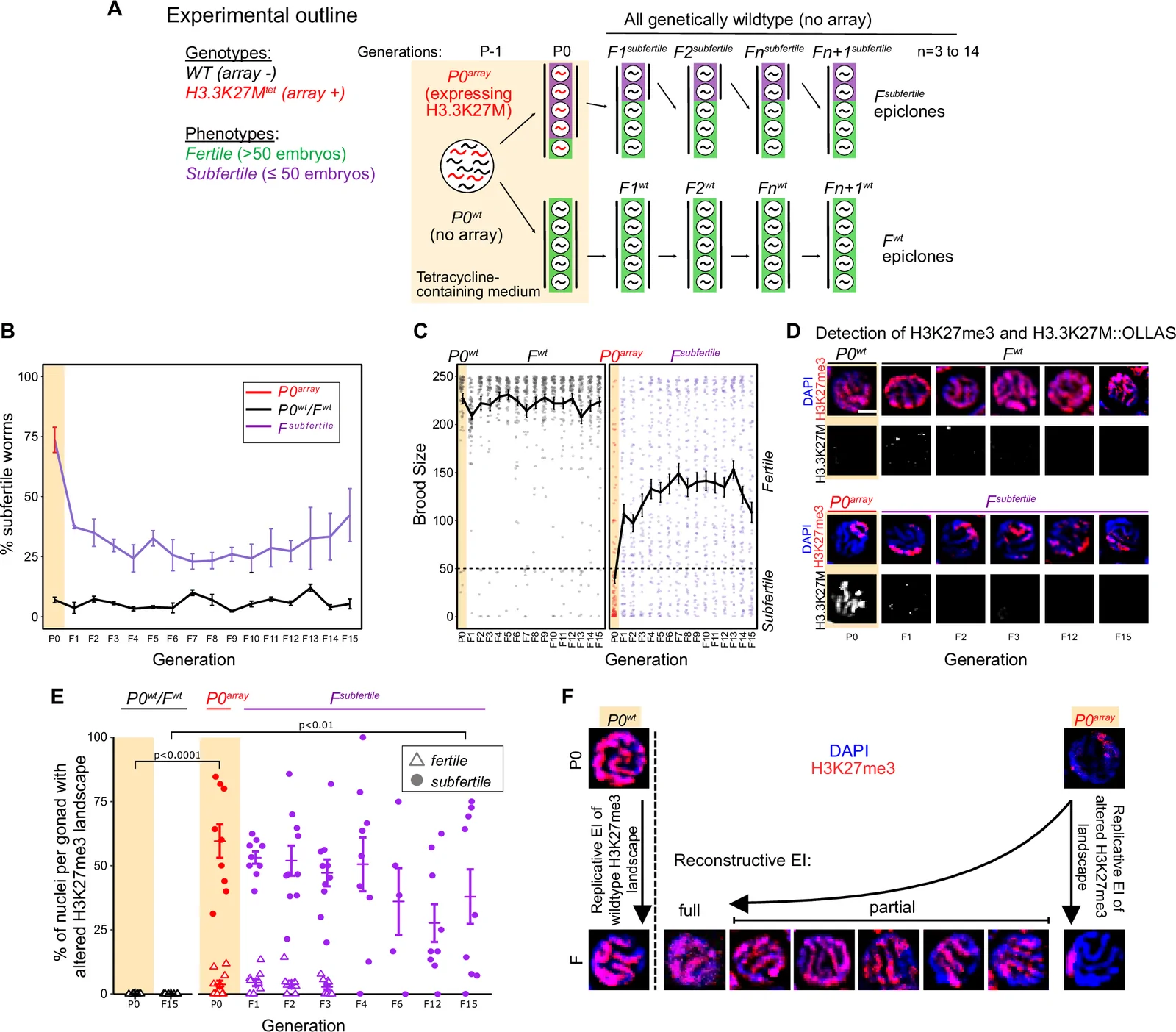
*H3.3K27M*^tet^-induced changes of the H3K27me3 landscape and the associated fertility defects are epigenetically inherited for many generations. (**A**) Experimental scheme for the study of transgenerational epigenetic inheritance of *H3.3K27M*^*tet*^-induced phenotypes. Animals carrying the *H3.3K27M*^*tet*^ extrachromosomal array are shown in red, while genetically wild-type animals are shown in black. Tetracycline-containing media are represented by a beige background in all panels. In the P-1 generation, wildtype and *H3.3K27M*^tet^ worms were transferred onto tetracycline-containing plates. In the P0 generation, worms carrying the *H3.3K27M*^tet^ array (*P0*^array^) were singled on tetracycline-containing plates, and were categorized into fertile (>50 embryos; green background) or subfertile (<50 embryos; purple background). Genetically wildtype F1 worms derived from subfertile* P0*^array^ worms were singled on plates without tetracycline. These *F1*^subfertile^ were again categorized into fertile or subfertile. The procedure was repeated until generation 15, with offspring of subfertile individuals from generation *Fn*^subfertile^ being singled to form the generation *Fn+1*^subfertile^. Wildtype worms (*P0*^wt^) and their offspring (*F*^wt^) worms were used as controls. The inheritance experiment was repeated three times with at least 50 individuals per phenotype in each generation. (**B**) Transgenerational infertility assay. Mean percentages of subfertile worms in *P0*^wt^/*F*^wt^ (black), *P0*^array^ (red), and *F*^subfertile^ (purple) in the generation shown on the x-axis label, from generation P0 to generation F15. Error bars indicate standard errors of three independent transgenerational assays, with on average 49 broods quantified per generation and condition (23 < *n* < 83 broods). (**C**) Brood size distribution of animals in *P0*^wt^/*F*^wt^ (black), *P0*^array^ (red), and *F*^subfertile^ (purple) from generation P0 to generation F15. Each dot represents the complete brood size of an individual worm in the generation shown on the x-axis label (23 < *n* < 85 brood sizes per generation and condition). Means are connected by black lines, and standard errors for each generation are shown. The dashed line demarks the arbitrary threshold between the fertile and subfertile phenotypes. Data for *P0*^wt^ and *P0*^array^ are the same as in (Fig. 1E). (**D**) Representative images from immunostaining of H3K27me3 (magenta) and OLLAS-tagged H3.3K27M (white) in pachytene nuclei counterstained with DAPI (blue) in generations P0, F1, F2, F3, F12, and F15 of *P0*^wt^*/F*^wt^ (top) and *P0*^array^*/F*^subfertile^ (bottom). Scale bar represents 2 µm. (**E**) Percentages of pachytene nuclei with altered H3K27me3 landscape per gonad in *P0*^wt^ *and F15*^wt^ and *P0*^array^*-F15*^subfertile^. Each data point represents the percentage of pachytene nuclei with altered H3K27me3 patterns within an individual gonad (at least 10 nuclei per gonad; 5 < *n* < 12 germ lines generations and conditions). Dots represent gonads from individual worms scored as subfertile, triangles represent gonads from individual worms scored as fertile in the generation shown on the x-axis label. The latter were only analyzed from *P0*^array^ to *F3*^subfertile^ generations. Means with standard errors of the data from subfertile individuals are shown. *P* values are from two-sided *t*-test comparisons (*p* = 1.7e^−5^ for P generation and *p* = 0.0074 for F generations). *F2-14*^wt^ generations were identical to *P0*^wt^ and *F15*^wt^. (**F**) Spectrum of the H3K27me3 landscapes in pachytene cells observed during reconstructive EI, with replicative EI of wildtype and *H3.3K27M*^tet^-induced altered landscapes shown for comparison. For quantifications of % nuclei with altered H3K27me3 landscapes per gonad in (**E**), only complete replicative EI was considered as “altered”, whereas full and partial reconstructive EI was considered as “wildtype”.

Next, we designed a transgenerational assay to investigate the transmission of the *H3.3K27M*^tet^-induced defects to wildtype offspring (Fig. 2A). We induced the expression of *H3.3K27M*^tet^ in the parental (P-1 and P0) generations, singled >50 array-containing individuals (*P0*^array^), and categorized them into fertile and subfertile according to their brood sizes. Among the offspring of the *P0*^array^ generation, we only selected offspring that had lost the *H3.3K27M*^tet^ array (genetically wildtype) and no longer expressed H3.3K27M. To assess the inheritance of fertility defects, we identified subfertile *P0*^array^ individuals (defined as ≤50 viable offspring) and singled >50 of their F1 offspring. When these *F1*^subfertile^ reached adulthood, we classified their fertility (fertile or subfertile). For each subsequent generation *Fn*^subfertile^, we again identified subfertile individuals, singled >50 of their offspring (the *Fn+1*^subfertile^), and classified the fertility (fertile or subfertile) of these offspring (Fig. 2A). Worms that did not contain the *H3.3K27M*^tet^ array in the P-1 and P0 generations and therefore never expressed H3.3K27M, as well as their offspring, were used as wildtype control (*P0*^wt^ and *F*^wt^). We singled all worms at larval stages before any germline or fertility phenotypes were visible to avoid observer bias.

In the P0 generation, 74% of the worms carrying the *H3.3K27M*^tet^ array (*P0*^array^) showed fertility defects, while less than 7% of the *P0*^wt^ worms were subfertile at 25 °C (Fig. 2B). Remarkably, the *H3.3K27M*^tet^-induced fertility defects were still present among the genetically wildtype F1 offspring of subfertile *P0*^array^ animals. 37% of these *F1*^subfertile^ inherited fertility defects (Fig. 2B). We expanded the TEI assays to generations F2 to F15 (Fig. 2A). We found that when selecting subfertile individuals in generation *Fn*^subfertile^, the fertility defects were present in 25–40% of their offspring in the generation *Fn+1*^subfertile^ (Fig. 2B). This demonstrates that under selection, the phenotypic consequences of the H3.3K27M expression in the P0 generation can be heritable for at least 15 generations in a population of wildtype worms. The offspring of subfertile worms displayed both subfertile and fertile phenotypes in each generation, implying that individual worms can both replicate the *H3.3K27M*^tet^-induced changes or reconstruct the wildtype H3K27me3 landscape, respectively, in different germ cells. The fertility defects among the *F*^wt^ (wildtype control) remained low and ranged between 3 and 12% for all generations analyzed (Fig. 2B).

To obtain a quantitative measure of the fertility defects, we plotted the brood sizes of the *P0*^wt^/*F*^wt^ and *P0*^array^/*F*^subfertile^ worms (Fig. 2C). Most *P0*^wt^ and *F*^wt^ animals had >200 offspring. *H3.3K27M*^tet^-induction resulted in a reduction of the brood size to less than 50 for 74% of the *P0*^array^ worms. The brood sizes of *F*^subfertile^ remained, on average, consistently lower compared to *F*^wt^ for at least 15 generations, with a large variability. This variability in brood sizes of the *F*^subfertile^ in every generation again implies that most worms inherit the fertility defects but are also able to reconstruct the wildtype H3K27me3 landscape in part of the germ cells, resulting in partially or fully recovered brood sizes.

**Figure EV1:**
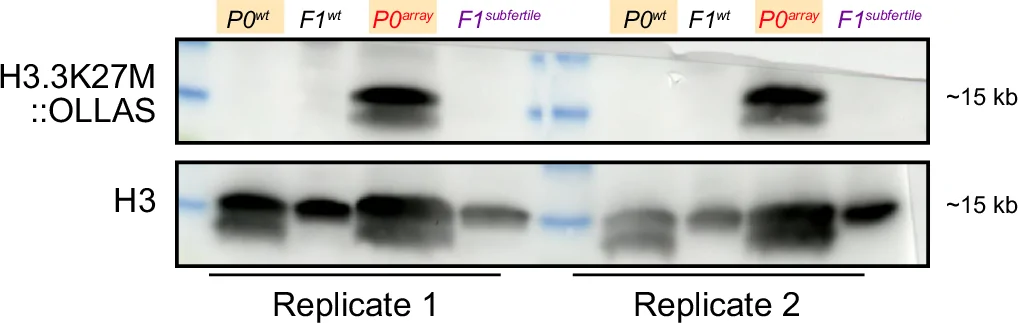
*H3.3K27M*^*tet*^ expression is restricted to P0. Detection of H3.3K27M protein by western blots in tetracycline-exposed *P0*^array^ and *F*^subfertile^, with *P0*^wt^ and *F1*^wt^ worms as controls. The OLLAS tag was used to detect H3.3K27M, and H3 was used as a loading control. Whole-worm lysates of 40 adult worms were loaded in each lane. A beige background signifies that the worms were grown on media containing 1 ng/µl tetracycline.

Next, we tested whether the inheritance of the fertility defects was based on the inheritance of the altered H3K27me3 landscape. We used IF to detect H3K27me3 in germ cells of *P0*^wt^ and *P0*^array^ and in *F*^wt^ and *F*^subfertile^ worms from generations F1 to F15. IF allows the detection of altered chromatin landscapes within single cells and is therefore ideally suited to probe patterns that are inherited with low penetrance and are heterogeneous within individuals. As described above (Fig. 1D), *H3.3K27M*^tet^-induced *P0*^array^ worms showed germ cells with an altered H3K27me3 landscape, where the H3K27me3 signal is depleted from autosomes but retained on chromosome X. Strikingly, the changed H3K27me3 landscape was present in all of the next 15 *F*^subfertile^ generations, even though the H3.3K27M protein was no longer detectable in any of the *F* generations (Figs. 2D and EV1). *F*^wt^ worms did not show altered H3K27me3 landscapes in any generation (Fig. 2D). These results demonstrate that both the altered H3K27me3 landscape and the resulting fertility defects induced by *H3.3K27M*^tet^ in the *P0* generation can be inherited for at least 15 generations in a genetically wildtype population.

We quantified the penetrance of the altered H3K27me3 landscape within individual worms, and assessed if a high penetrance of the chromatin defects resulted in a low brood size of that individual. We estimated whether individual *P0*^wt^*/F*^wt^ or *P0*^arra*y*^*/F*^subfertile^ animals were fertile or subfertile by letting them lay eggs until the second day of adulthood, and then analyzed the H3K27me3 patterns of the germ cells of these same individuals by IF. To quantify the penetrance of the chromatin changes, we determined the percentage of nuclei with clearly recognizable altered H3K27me3 patterns per gonad (Fig. 2E). Most *P0*^array^*/F*^subfertile^ individuals contained a mix of germ cells with altered H3K27me3 landscapes and with fully or partially reconstructed wildtype-like H3K27me3 landscapes (Fig. 2F). For the quantification of the penetrance, we only counted nuclei with completely aberrant landscapes as “altered”, thus likely underestimating the penetrance of the defects. *P0*^wt^ and *F*^wt^ individuals did not show any altered H3K27me3 patterns and faithfully replicated the wildtype H3K27me3 landscapes (Fig. 2E). Germ lines of *P0*^array^ animals classified as subfertile (brood size <50) contained on average 60% nuclei with altered patterns (Fig. 2E). Strikingly, the *P0*^array^ individuals that were classified as fertile (“escapers”; brood size >50) had on average only 4% nuclei with altered H3K27me3 patterns, confirming a causal link between altered H3K27me3 patterns and reduced fertility. Consistent with a robust epigenetic inheritance, we observed germ cells with altered H3K27me3 patterns with a penetrance of on average 30–50% in subfertile individuals of the *F1*^subfertile^ to *F15*^subfertile^ generations. Some *F*^subfertile^ individuals still showed 75% germ cells with altered H3K27me3 landscapes in generation F15, demonstrating that they can be robustly transmitted across generations (Fig. 2E). Germ lines of *F*^subfertile^ individuals classified as subfertile contained more nuclei with altered patterns than individuals classified as fertile (the latter were only assessed in F1–F3). The partial penetrance of the altered H3K27me3 patterns within individual germ lines explains how each individual in each generation can give rise to both fertile and subfertile offspring. The variability of the H3K27me3 patterns both between and within individuals also emphasizes the need for methods that allow the assessment of individual worms and cells (such as IF) instead of methods that average the signals of many cells from many individuals (such as ChIP-seq).

Taken together, our observations imply that during the transition from one *F*^subfertile^ generation to the next, some germ cells reconstruct the wildtype H3K27me3 landscape and give rise to fertile offspring, while other germ cells replicate the altered H3K27me3 patterns and give rise to subfertile offspring. When selecting for subfertile individuals, the *H3.3K27M*^tet^-induced chromatin changes can be epigenetically inherited for at least 15 generations in a population of genetically wild-type worms.

### Downstream effects of altered H3K27me3 patterns persist during TEI

**Figure 3:**
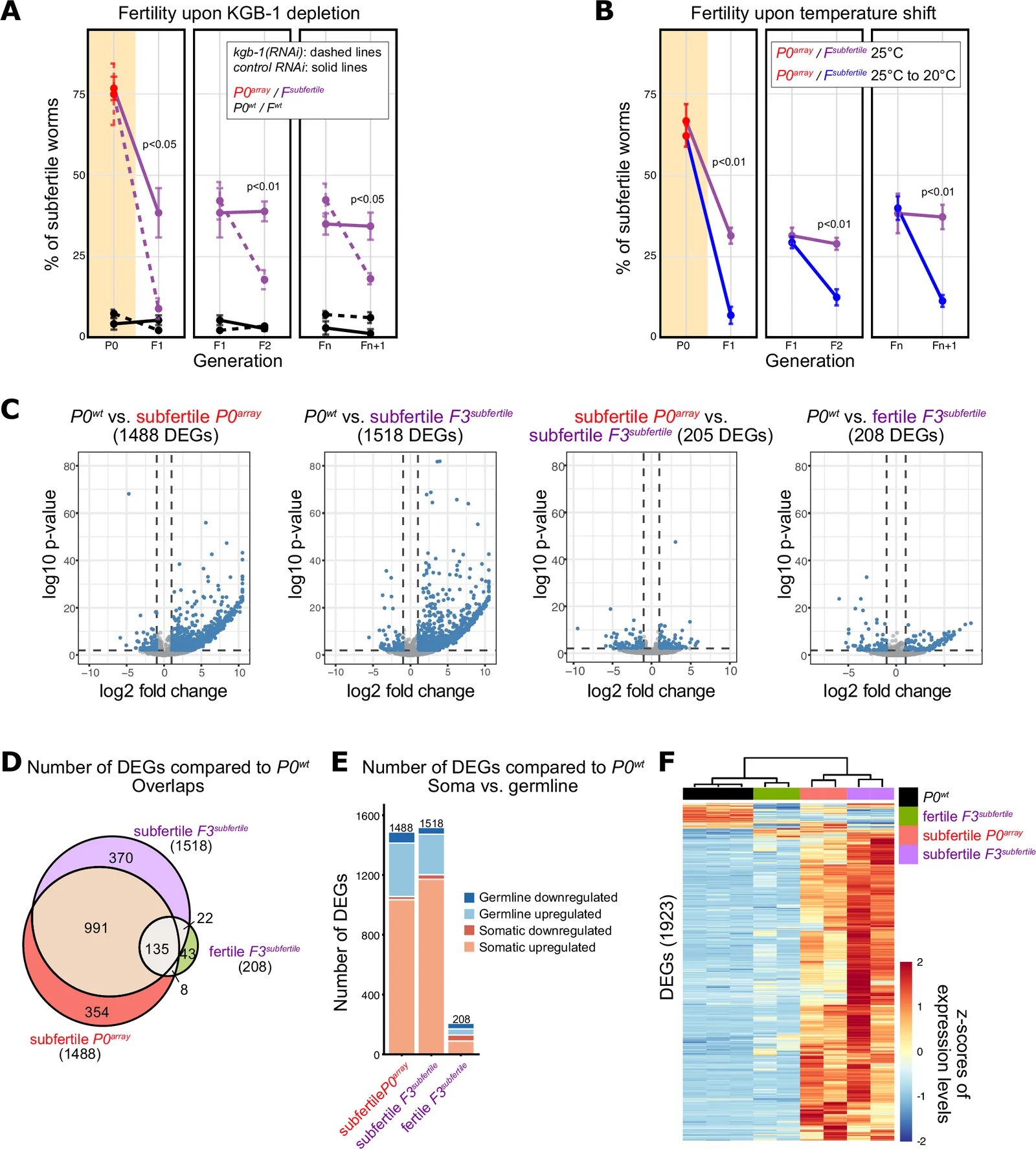
The downstream effects of altered H3K27me3 patterns are maintained during TEI. (**A**) Transgenerational infertility assay upon *kgb-1(RNAi)* treatment. Mean percentages of subfertile worms in *P0*^wt^ and *F*^wt^ (black), and *P0*^array^ (red) and *F*^subfertile^ (purple) are shown. Animals were exposed to *kgb-1(RNAi)* treatment (dashed lines) from P0 to F1 (left), or from F1 to F2 (middle), or from F2-F3, F3-F4, and F4-F5 (right; data pooled as Fn), and fertility of the following generation was assessed. Animals were exposed to *control RNAi* treatment (solid lines) in parallel. Means and standard deviations of three independent transgenerational assays are shown, with 26 < *n* < 58 broods analyzed per generation and condition. 2-sided *t*-tests were used to test for the significance of the difference between the values of the *F*^subfertile^ in the inheriting generation with and without *kgb-1(RNAi)* treatment (*p* = 0.044 for P0 to F1, *p* = 0.0077 for F1 to F2, *p* = 0.045 for Fn to Fn + 1). (**B**) Transgenerational infertility assay upon temperature shift from 25 °C to 20 °C. Mean percentages of subfertile worms in *P0*^a^^rray^ (red) and *F*^subfertile^ (purple, maintained at 25 °C; blue, shifted from 25 °C to 20 °C) are shown. Animals were moved from 25 °C to 20 °C at P0 (left), or F1 (middle), or F2, F3, or F4 (right; data pooled as Fn), and fertility of the following generation was assessed (blue lines). Animals were maintained at 25 °C in parallel (purple lines). Means and standard deviations of at least three independent transgenerational assays are shown, with 30 < *n* < 55 broods analyzed per generation and condition. Two-sided *t*-tests were used to test for the significance of the difference between the values of the *F*^subfertile^ in the inheriting generation with and without temperature shift (*p* = 0.0013 for P0 to F1, *p* = 0.0061 for F1 to F2, *p* = 0.0024 for Fn to Fn + 1). (**C**–**F**) RNA-seq analysis of dissected germ lines from *P0*^wt^ (wildtype control; *n* = 3), subfertile individuals from *P0*^array^ (*n* = 2), subfertile individuals from *F3*^subfertile^ (*n* = 2), and fertile individuals from *F3*^subfertile^ (*n* = 2). Each sample contains a pool of about 20 dissected germ lines. (**C**) Volcano plots for pairwise comparison of the indicated samples. Differentially expressed genes (DEGs) were identified based on a fold change of ≥2 and an FDR of ≤0.01 and are shown in blue. The Wald test was used to compute *p* values. (**D**) Venn diagram showing the overlap of DEGs in subfertile individuals from *P0*^array^, subfertile individuals from *F3*^subfertile^, and fertile individuals from *F3*^subfertile^. (**E**) Classification of DEGs into germline or somatic genes and up- or downregulation. (**F**) Heatmap showing z-scores of expression levels of all the genes that were differentially expressed in one of the samples. Unbiased clustering grouped the samples with the highest similarities.

In our previous analysis of a strain with constitutive expression of H3.3K27M, we had shown that the loss of autosomal H3K27me3 results in ectopic activation of many genes that are normally silenced in the germline. This resulted in fertility defects at 25 °C that could be suppressed by the depletion or inhibition of KGB-1, or by growing the worms at 20 °C (Delaney et al, 2019). Given that we observed an inheritance of the changed H3K27me3 chromatin patterns in our TEI assay, we hypothesized that the fertility defects in the inheriting generations should also depend on the expression of *kgb-1* and the growth at 25 °C. To test this hypothesis, we depleted KGB-1 by RNAi from *P0*^array^ to *F1*^subfertile^, or in the subfertile generations (F1 to F2, F3 to F4, or F4 to F5) (Fig. 3A). In a parallel experiment, we shifted the worm cultures from 25 °C to 20 °C in the same generations as for the KGB-1 depletion experiment (Fig. 3B). Both depletion of KGB-1 and a temperature shift to 20 °C suppressed the fertility defects in the subsequent *F*^subfertile^ generations (Fig. 3A,B). These results demonstrate that the fertility defects can be suppressed by the same treatment in both the *P0*^array^ and the *F*^subfertile^, supporting the notion that the underlying cause of the phenotype remains the same during TEI.

**Figure EV2:**
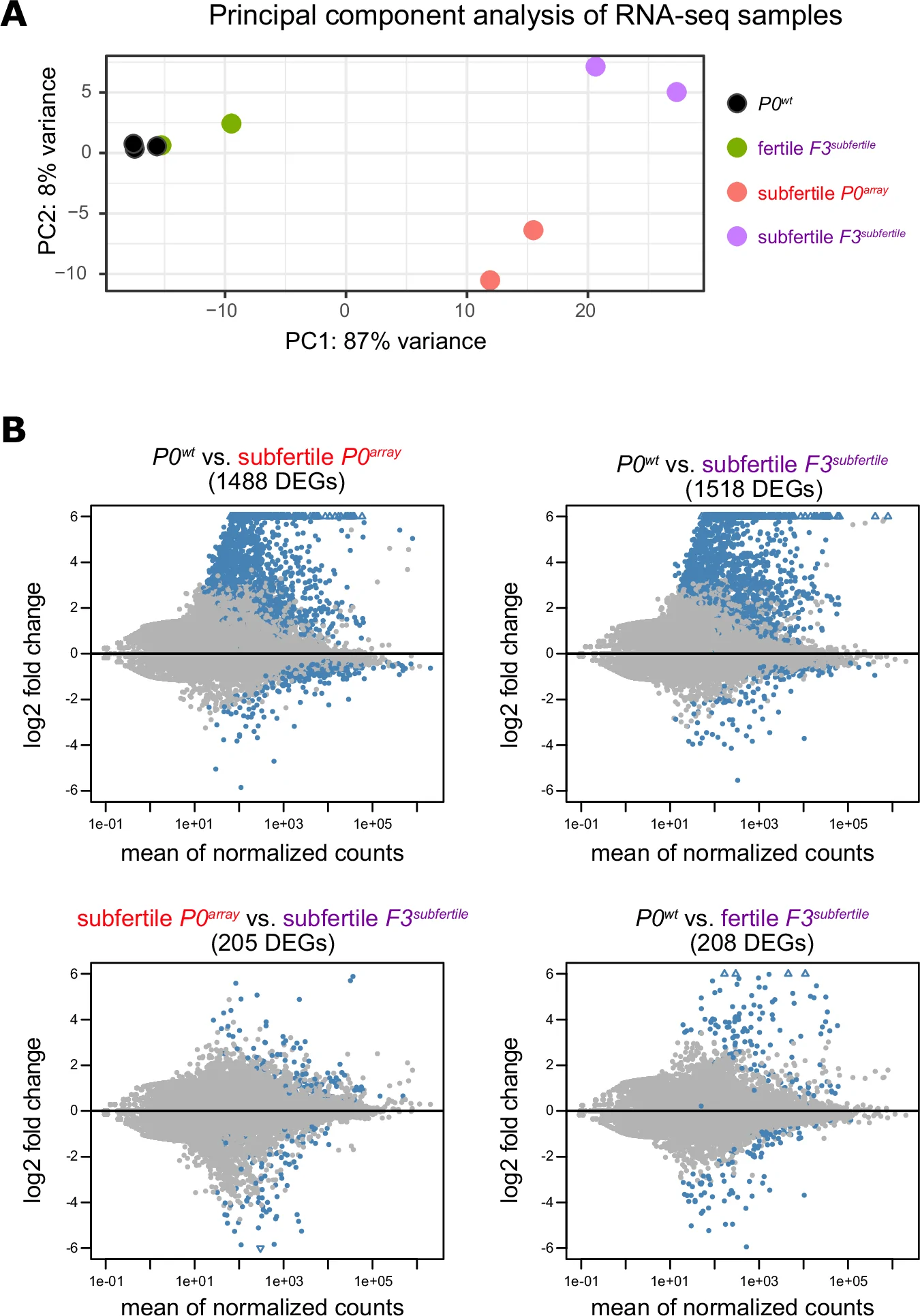
RNA-seq analysis of germ lines in the parental and inheriting generations. Germ lines were dissected from *P0*^wt^ (wildtype control; *n* = 3), subfertile individuals from *P0*^array^ (*n* = 2), subfertile individuals from *F3*^subfertile^ (*n* = 2), and fertile individuals from *F3*^subfertile^ (*n* = 2). Each sample contained a pool of about 20 dissected germ lines. (**A**) PCA analysis of the RNA-seq samples, showing that biological replicates cluster together, and samples from fertile individuals are distinct from samples from subfertile individuals. (**B**) Volcano plots for pairwise comparison of the indicated samples. Differentially expressed genes (DEGs) were identified based on a fold change of ≥2 and an FDR of ≤0.01 and are shown in blue.

To assess whether the inheritance of the changed chromatin patterns resulted in similar patterns of aberrant gene expression in the *P0*^array^ and the *F*^subfertile^ generations, we dissected germ lines of adult individuals in the P0 and F3 generations and analyzed their gene expression by RNA-seq (Figs. 3C–F and EV2A,B). We compared fertile individuals from *P0*^wt^ (wildtype control), subfertile individuals from *P0*^array^, subfertile individuals from *F3*^subfertile^, and fertile individuals from *F3*^subfertile^. We pooled 20 dissected germ lines per sample, and compared the *P0*^array^ and *F3*^subfertile^ samples to the *P0*^wt^ sample. The RNA-seq analysis showed that the induced changes of the H3K27me3 landscape led to the ectopic expression of 1488 genes in the germ lines of *P0*^array^ worms (Fig. 3C,D and EV2A,B). 1518 genes were still ectopically expressed in subfertile worms of the *F3*^subfertile^ generation, with an overlap of 1126 genes with the *P0*^array^ (Fig. 3C,D). The sets of ectopically expressed genes for both the *P0*^array^ and subfertile individuals from the *F3*^subfertile^ were enriched for somatic genes (Fig. 3E), consistent with the ectopic de-silencing of somatic genes in the germ line upon loss of H3K27me3. The results demonstrate that the chromatin-based inheritance leads to an inheritance of the same downstream effects that were induced in the *P0*^array^ generation. Worms that had recovered their fertility to wildtype levels among the *F3*^subfertile^ showed gene expression patterns that are very similar to wildtype worms (*P0*^wt^) (Figs. 3C–F and EV2A,B), further demonstrating that changed H3K27me3 patterns, aberrant gene expression and fertility defects are linked. Although *kgb-1* showed a trend towards upregulation in the *P0*^array^ (fold change 2.3; FDR 0.15) and subfertile individuals of the *F3*^subfertile^ generation (fold change 2.3; FDR 0.43) samples, it was not among the significantly upregulated genes based on our cutoffs (fold change ≥2; FDR of ≤0.01). The RNA-seq analysis nevertheless integrates the chromatin changes and the fertility defects, and provides a molecular link between H3K27me3 patterns, gene expression and fertility during transgenerational epigenetic inheritance.

### Spreading of H3K36me3 promotes TEI of altered H3K27me3 patterns

Next, we wondered what prevented the reconstruction of wildtype H3K27me3 patterns in the offspring that inherited the changed H3K27me3 distribution. The histone PTM H3K36me3 has previously been shown to antagonize H3K27me3 in the *C. elegans* germ line. H3K27me3 and H3K36me3 form mutually exclusive domains across the genome of germ cells to allow the expression of germline genes and to repress somatic genes, respectively (Gaydos et al, 2012). We therefore hypothesized that genomic regions that lose H3K27me3 upon expression of H3K27M gain ectopic H3K36me3. These altered H3K36me3 epialleles may then antagonize the reconstruction of wild-type H3K27me3 epialleles during TEI.

**Figure 4:**
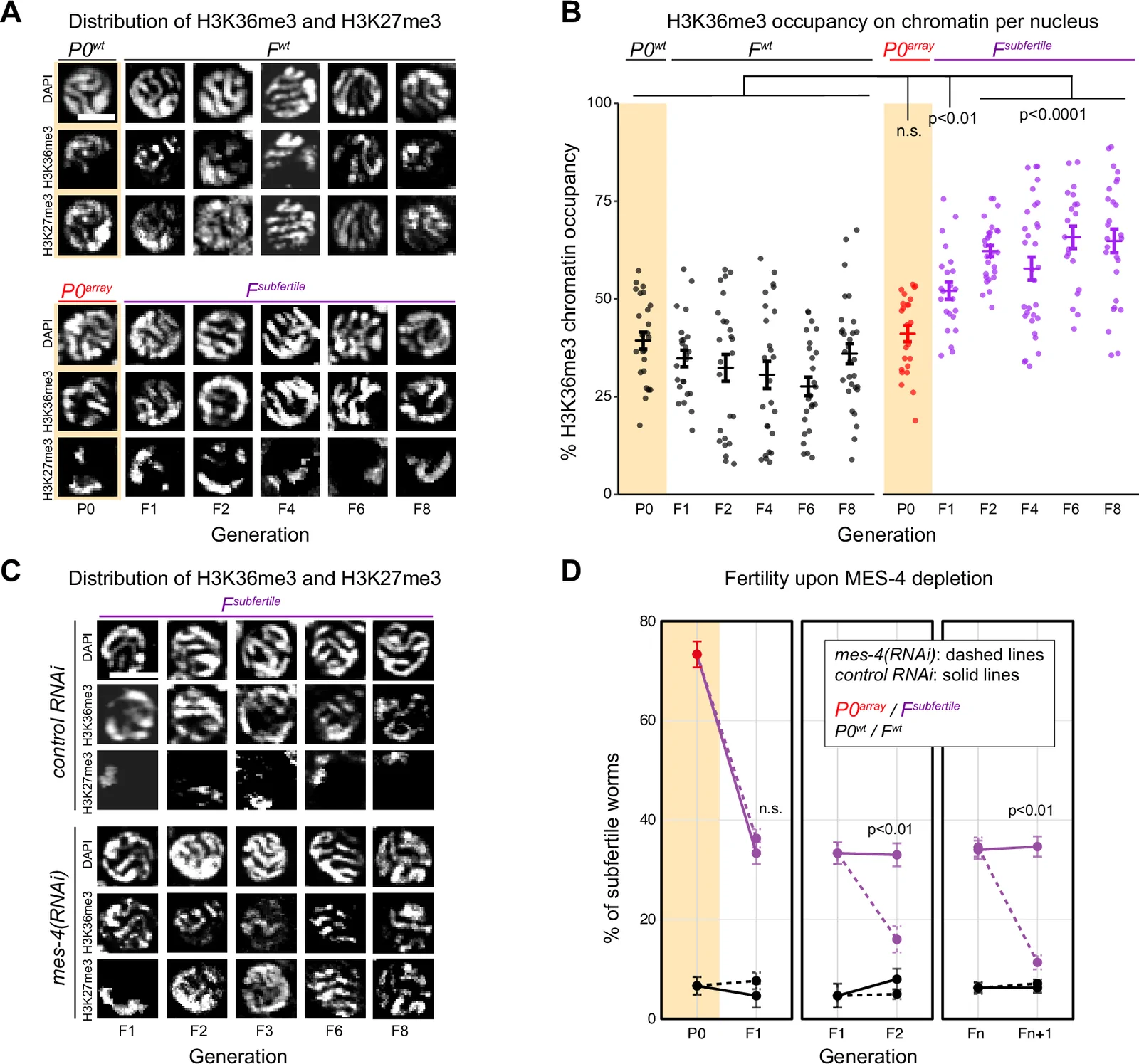
Spreading of H3K36me3 promotes TEI of altered H3K27me3 patterns in generations after *F1*^*subfertile*^. (**A**) Representative images from immunostaining of H3K27me3 and H3K36me3 in pachytene nuclei counterstained with DAPI in generations *P0*^wt^*-F8*^wt^ (top) and *P0*^array^*-F8*^subfertile^ (bottom). Scale bar represents 4 µm. Generations grown on tetracycline-containing media are indicated by a beige background in all panels. (**B**) Quantification of H3K36me3 chromatin occupancy in generations *P0*^wt^*-F8*^wt^ (black), *P0*^array^ (red), and *F1-F8*^subfertile^ (purple). Each dot represents the percent of chromatin with H3K36me3 signal per nucleus. At least 20 nuclei (20 < *n* < 31) were scored per generation, and means with standard errors are shown. *P* values are from two-way ANOVA with Sidak’s multiple comparison tests, comparing the indicated *P0*^array^*/F*^subfertile^ generations with the corresponding *P0*^wt^*/F*^wt^ generations (n.s. stands for not significant; *p* = 0.58 for *P0*^array^, *p* = 0.008 for *F1*^subfertile^, *p* = 2.2e^−16^ for *F2-8*^subfertile^). (**C**) Representative images from immunostaining of H3K27me3 and H3K36me3 in pachytene nuclei counterstained with DAPI in *F1-F8*^subfertile^ generations treated with control* RNAi* (top) or *mes-4(RNAi)* (bottom). Scale bar represents 4 µm. (**D**) Transgenerational infertility assay upon *mes-4(RNAi)* treatment. Mean percentages of subfertile worms in *P0*^wt^ and *F*^wt^ (black), and *P0*^array^ (red) and *F*^subfertile^ (purple) are shown. Animals were exposed to *mes-4(RNAi)* treatment (dashed lines) in P0 (left), F1 (middle), or F2, F5, F6, or F7 (right; data pooled as Fn), and fertility of the following generation was assessed. Animals were exposed to control* RNAi* treatment (solid lines) in parallel. Means and standard deviations of four independent transgenerational assays are shown, with 21 < *n* < 46 broods analyzed per generation and condition. Two-sided *t*-tests were used to test for the significance of the difference between the values of the *F*^subfertile^ in the inheriting generation with and without *mes-4(RNAi)* treatment (n.s. stands for not significant; *p* = 0.35 for P0 to F1, *p* = 0.008 for F1 to F2, *p* = 0.005 for Fn to Fn + 1).

We co-stained H3K36me3 and H3K27me3 in pachytene nuclei and quantified the H3K36me3 chromatin occupancy by comparing the H3K36me3 signal with the DAPI signal. This analysis revealed an increased signal of H3K36me3 in *F*^*subfertile*^ pachytene nuclei where H3K27me3 was reduced (Fig. 4A,B). However, the increase in H3K36me3 was not observed in the *P0*^*array*^ generation (Fig. 4A,B). These results are consistent with an autosomal replacement of H3K27me3 with H3K36me3 in pachytene nuclei of *F*^subfertile^, but imply that during the transition from P0 to F1, the spreading of H3K36me3 is not involved in initiating the inheritance. As expected, no H3K36me3 changes were observed in *P0*^wt^*/F*^wt^, where all the nuclei have a wild-type H3K27me3 distribution (Fig. 4A,B).

**Figure EV3:**
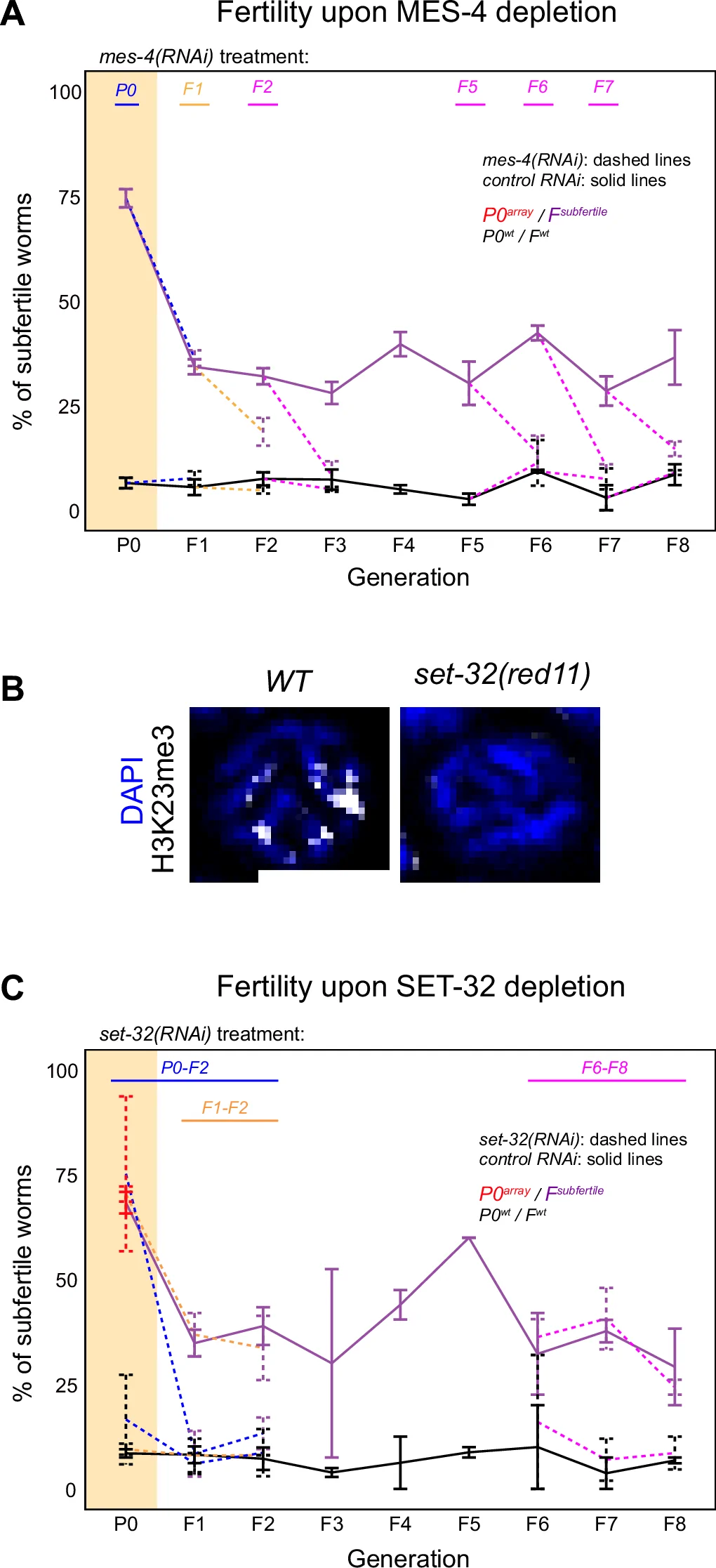
Transgenerational infertility assay upon HMT depletion. (**A**) Transgenerational infertility assay upon *mes-4(RNAi)* treatment. Mean percentages of subfertile worms in generations *P0*^wt^*-F8*^wt^ (black, and dashed lines) and *P0*^array^*-F8*^subfertile^ (purple, and dashed lines). Animals were exposed to *mes-4(RNAi)* treatment (dashed lines) in P0 (blue), F1 (orange), F2, F5, F6, or F7 (fuchsia), and the fertility of the following generation was assessed. Animals were exposed to control RNAi treatment (solid lines) in parallel. Means with standard errors of at least two independent transgenerational assays are shown. (**B**) SET-32 is the main writer for H3K23me3 in pachytene nuclei. Representative images from immunostaining of H3K23me3 (white) in pachytene nuclei of wild-type and *set-32(red11)* animals grown at 25 °C. Scale bar represents 4 µm. (**C**) Transgenerational infertility assay upon *set-32(RNAi)* treatment. Mean percentages of subfertile worms in generations *P0*^wt^*-F8*^wt^ (black and dashed lines) and *P0*^array^*-F8*^subfertile^ (purple and dashed lines). Animals were exposed to *set-32(RNAi)* treatment (dashed lines) from P0 to F2 (blue), or from F1 to F2 (orange), or from F6 to F8 (fuchsia). Animals were exposed to control RNAi treatment (solid lines) in parallel. Means and standard errors of at least three independent transgenerational assays are shown.

To test if the spreading of H3K36me3 is required for the replicative TEI of the *H3.3K27M*^tet^-induced defects, we depleted MES-4, the histone methyltransferase required for the maintenance of H3K36me3 (Gaydos et al, 2012; Rechtsteiner et al, 2010; Furuhashi et al, 2010), during the transition from one generation to the next. Loss of MES-4 results in a maternal-effect sterile (Mes) phenotype (Furuhashi et al, 2010), and we could therefore only analyze the generation immediately following the start of the *mes-4(RNAi)* treatment. *Mes-4(RNAi)* treatment of the *P0*^wt^*/F*^wt^ had no effect on the fertility of the following generation, but maintaining the RNAi-mediated depletion of MES-4 for two generations resulted in the expected Mes phenotype with >90% penetrance, demonstrating that the depletion was efficient. Depletion of MES-4 reduced, but did not completely abolish H3K36me3 signal in pachytene nuclei (Fig. 4C), likely because of the activity of other H3K36 HMTs such as MET-1 (Kreher et al, 2018; Cockrum and Strome, 2022). When we started the exposure to *mes-4(RNAi)* in generation *F1*^subfertile^ or in later generations (*Fn*^subfertile^, with *n* = 2, 5, 6, or 7), we observed a full suppression of the replicative EI of the *H3.3K27M*^tet^-induced defects, and worms of the subsequent generation showed H3K27me3 landscapes and fertility comparable to wildtype controls (Figs. 4C,D and EV3A). These results suggest that in the absence of MES-4, the lower levels of H3K36me3 allow the reconstruction of wild-type H3K27me3 domains during the development of the subsequent generation, thus restoring fertility. They also demonstrate that the ectopic H3K36me3 epialleles formed upon redistribution of H3K27me3 are required for the replicative TEI of the altered H3K27me3 epialleles and the resulting fertility defects. Surprisingly, starting the *mes-4*(*RNAi)* treatment at *P0*^array^ generation had no suppressive effect on the inherited fertility defects or on the altered H3K27me3 landscape in *F1*^subfertile^ worms (Fig. 4C,D). These results suggest that MES-4 is required for maintaining the ectopically initiated H3K36me3 epialleles in *F*^subfertile^, but not for initiating the TEI during the transition from *P0*^array^ to *F1*^subfertile^. We therefore hypothesized that another histone PTM is required for the initiation of TEI of the altered H3K27me3 epialleles.

### Spreading of H3K23me3 is required for the initiation of TEI of altered H3K27me3 patterns

**Figure 5:**
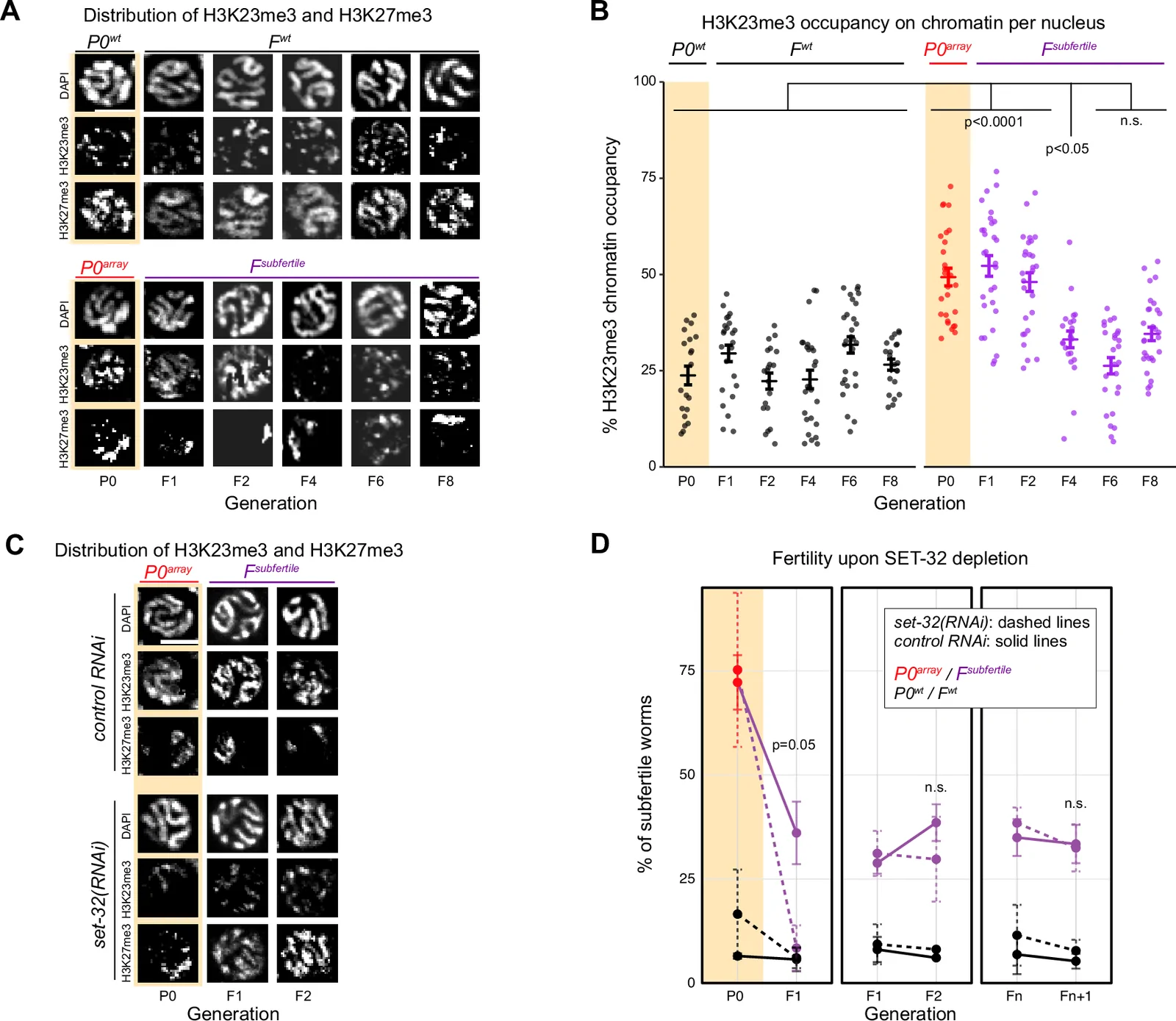
Spreading of H3K23me3 is required for the initiation of TEI of altered H3K27me3 patterns. (**A**) Representative images from immunostaining of H3K27me3 and H3K23me3 in pachytene nuclei counterstained with DAPI, in generations *P0*^wt^*-F8*^wt^ (top) and *P0*^array^*-F8*^subfertile^ (bottom). Scale bar represents 4 µm. Generations grown on tetracycline-containing media are indicated by a beige background in all panels. (**B**) Quantification of H3K23me3 chromatin occupancy in generations *P0*^wt^*-F8*^wt^ (black), *P0*^array^ (red), and *F1-F8*^subfertile^ (purple). Each dot represents the percent of chromatin with H3K23me3 signal per nucleus. At least 20 nuclei (26 < *n* < 29) were scored per generation, and means with standard errors are shown. *P* values are from two-way ANOVA with Sidak’s multiple comparison tests, comparing the indicated *P0*^array^*/F*^subfertile^ generations with the corresponding *P0*^wt^*/F*^*wt*^ generations (n.s. stands for not significant; *p* = 1.8e^−10^ for *P0*^array^-*F2*^subfertile^, *p* = 0.035 for *F4*^subfertile^, *p* = 0.18 for *F6-F8*^subfertile^). (**C**) Representative images from immunostaining of H3K27me3 and H3K23me3 in pachytene nuclei of *P0*^array^*-F2*^subfertile^ generations treated with control* RNAi* (top) or *set-32(RNAi)* (bottom). Scale bar represents 4 µm. (**D**) Transgenerational infertility assay upon *set-32(RNAi)* treatment. Mean percentages of subfertile worms in *P0*^wt^ and *F*^wt^ (black), and *P0*^array^ (red) and *F*^subfertile^ (purple) are shown. Animals were exposed to *set-32(RNAi)* treatment (dashed lines) from P0 to F1 (left), or from F1 to F2 (middle), or from F5 to F7 (right; data pooled as Fn), and fertility of the following generation was assessed. Animals were exposed to control* RNAi* treatment (solid lines) in parallel. Means and standard deviations of three independent transgenerational assays are shown, with 25 < *n* < 53 broods analyzed per generation and condition. Means and standard deviations of at least three biological replicates are shown. Two-sided *t*-tests were used to test for the significance of the difference between the values of the *F*^subfertile^ in the inheriting generation with and without *set-32(RNAi)* treatment (n.s. stands for not significant; *p* = 0.017 for P0 to F1, *p* = 0.49 for F1 to F2, *p* = 0.90 for Fn to Fn + 1).

Previous studies implicated the methyltransferase SET-32 in the initiation of small-RNA-mediated epigenetic inheritance (Woodhouse et al, 2018; Spracklin et al, 2017). SET-32 catalyzes the methylation of H3K23, and no H3K23me3 is observed in pachytene nuclei in *set-32* mutants, indicating that SET-32 is the main HMT for H3K23me3 at the pachytene stage of the *C. elegans* germ line (Fig. EV3B). We hypothesized that H3K23me3 replaces H3K27me3 upon *H3K27M*^tet^-induction and initiates EI of the altered epialleles. To test this hypothesis, we detected H3K23me3 by IF from P0 to F8 in *P0*^wt^*/F*^wt^ and *P0*^array^*/F*^subfertile^ animals, and quantified the H3K23me3 chromatin occupancy by comparing the H3K23me3 signal with the DAPI signal. In *P0*^wt^/*F*^wt^ animals, H3K23me3 appeared as localized spots on chromatin, consistent with previous observations in wildtype worms (Vandamme et al, 2015) (Fig. 5A). Upon the induction of *H3.3K27M*^tet^ in the *P0*^array^ animals, the H3K23me3 signal increased and appeared as a continuous pattern on the chromosomes in *P0*^array^ nuclei with altered H3K27me3 landscapes. The increase in H3K23me3 persisted in the *F1-F2*^subfertile^ generations, but subsequently returned to wildtype levels and distributions after *F2*^subfertile^ (Fig. 5A,B). These results suggest that H3K23me3 replaces H3K27me3 in genomic regions where it is lost upon *H3.3K27M*^tet^ induction, initiating the EI of the altered H3K27me3 patterns. However, the increase of H3K23me3 is transient, and is lost and likely replaced by H3K36me3 in later generations.

We next examined whether the increased deposition of H3K23me3 is required for the replicative TEI of the *H3.3K27M*^tet^-induced phenotypes, and whether it could bridge the reduction of H3K27me3 and the increase of H3K36me3 during the *P0*^array^ to *F1*^subfertile^ transition. We hypothesized that the expression of *H3.3K27M*^tet^ induces the SET-32-mediated deposition of H3K23me3 in the *P0*^array^ generation, and that the ectopic H3K23me3 epialleles counteract the reconstructive EI of wildtype H3K27me3 domains during the transition to the *F1*^subfertile^ generation. To test this hypothesis, we depleted SET-32 by RNAi from P0 to F1 and examined the inheritance of *H3.3K27M*^tet^-induced fertility defects. Depletion of SET-32 did not affect fertility in the *P0*^array^ (nor in the *P0*^wt^ or *F*^wt^). However, it fully suppressed the *H3K27M*^tet^*-*induced fertility defects and restored the wildtype H3K27me3 landscape in *F1*^subfertile^ generations (Figs. 5C,D and EV3C). In contrast, depleting SET-32 in the F generations, either during *F1-F2*^subfertile^ or during *F5-F7*^subfertile^ transitions, did not suppress the fertility defects or their inheritance to the subsequent generations (Fig. 5D). These results demonstrate that SET-32 plays a major role in the intergenerational inheritance of *H3K27M*^tet^-induced defects from *P0*^array^ to *F1*^subfertile^ generations, and in initiating TEI. They suggest that SET-32 deposits H3K23me3 on autosomal regions where H3K27me3 is lost in the *P0*^array^ generation. During the transitions from *F1-F2*^subfertile^ generations, H3K23me3 is then replaced by H3K36me3.

**Figure 6:**
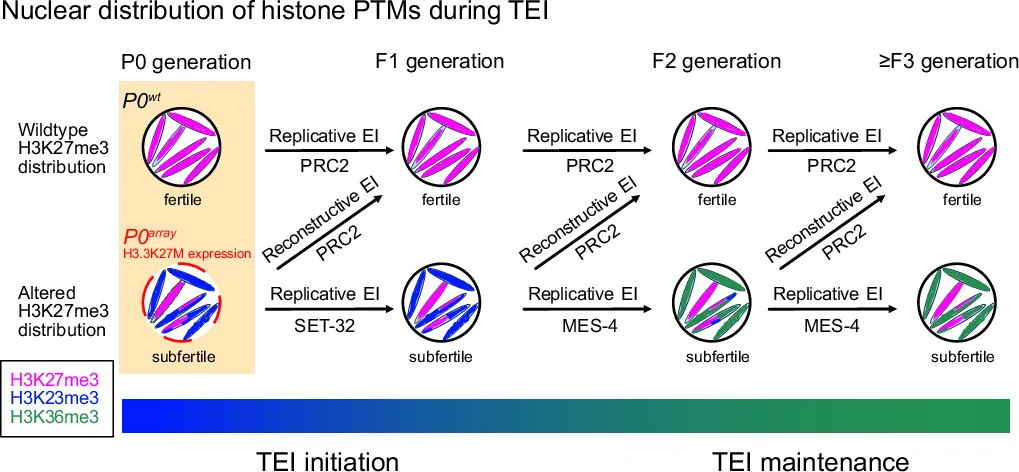
Model for the inheritance of altered H3K27me3 patterns. The distribution of H3K27me3 (magenta) in wild-type pachytene nuclei and upon H3.3K27M-mediated redistribution are shown. For simplicity, H3K23me3 (blue) and H3K36me3 (green) are only shown when their levels are increased and spread. H3K27me3 is distributed over all chromosomes in wildtype. In the *P0*^array^ generation, induction of H3.3K27M results in the displacement of PRC2 and a restriction of the H3K27me3 signal to the X chromosome and a few autosomal domains. In the F generations, after loss of the H3.3K27M protein, there are two inheritance paths of the epialleles to the subsequent generations: replicative EI, where the altered H3K27me3 patterns are inherited to the next generation, and reconstructive EI, where the wildtype patterns are restored during the transition to the next generation. Replicative EI is divided into an initiation and a maintenance phase. During the initiation phase from P0 to F1/F2, SET-32 transiently spreads H3K23me3, which is required for the EI of the altered H3K27me3 patterns from P0 to F1. During the maintenance phase from F1/F2 to later generations, H3K36me3 replaces H3K23me3, and the MES-4-mediated deposition of H3K36me3 is required for the maintenance of the altered H3K27me3 patterns. In every generation, part of the germ cells switches to reconstructive EI, where PRC2 restores a wild-type H3K27me3 distribution. The different H3K27me3 distributions are linked to fertility phenotypes, as subfertile animals contain germ cells with altered H3K27me3 landscapes, whereas fertile animals only contain germ cells with wild-type H3K27me3 landscapes.

Based on these observations, we propose a model for the TEI of altered H3K27me3 domains. In the *P0*^array^, *H3.3K27M*^tet^ expression causes a depletion of autosomal H3K27me3 domains. SET-32 methylates H3K23 in the genomic regions where H3K27me3 is lost, which is required for the EI of the altered H3K27me3 patterns to the F1 generation (initiation phase). During the transition from F1/F2 to the later F generations, H3K36me3 replaces H3K23me3 and is required for the maintenance of the altered H3K27me3 patterns. During the maintenance phase, some germ cells can reconstruct wild-type H3K27me3 epialleles through the de novo writer activity of PRC2. In other germ cells, MES-4 maintains the ectopic H3K36me3 epialleles, thus antagonizing the reconstruction of wild-type H3K27me3 domains (Fig. 6). This model separates the TEI of H3K27me3 patterns into an initiation and a maintenance phase, and explains the balance of replicative and reconstructive TEI mediated by the HMTs PRC2 and MES-4 across generations (Fig. 6).

## Discussion

In this study, we demonstrate the TEI of an altered H3K27me3 landscape for many generations in a wild-type *C. elegans* population. Previous studies that have investigated EI have focused on the heritable (de)silencing of reporter transgenes by an acutely applied environmental stress (Klosin et al, 2017) or exogenously supplied small RNAs (Buckley et al, 2013; Priyadarshini et al, 2022; Ni et al, 2014; Ashe et al, 2012; Houri-Ze’evi et al, 2016; Lev et al, 2017). These studies have demonstrated that the effects on gene expression states can be passed on to unexposed offspring across several generations, requiring the RNAi machinery and involving chromatin modifications associated with gene silencing (Buckley et al, 2013; Demoinet and Roy, 2018). In parallel, studies describing the genetic removal of chromatin readers and writers provided insight into how chromatin marks are deposited and maintained (Stewart-Morgan et al, 2020; Greer et al, 2014; Heestand et al, 2018; Kaneshiro et al, 2022). Together, these studies clearly demonstrated the existence of TEI in *C. elegans*, but left open the questions of whether TEI can rely on histone PTMs as inheriting factors, and how the changes in chromatin were maintained across generations. Here, we revealed that an altered H3K27me3 landscape and the associated fertility defects can be transgenerationally heritable. Visualization of the H3K27me3 redistribution by IF allowed us to determine the persistence and penetrance of the inheritance in individual worms. We found that the TEI comprises two distinct steps: initiation, and maintenance (Fig. 6). In the initiation phase, the reduction of H3K27me3 results in an increase in SET-32-mediated deposition of H3K23me3. During the transition from initiation to maintenance (F1 to F2 generations), H3K23me3 levels diminish and MES-4-mediated deposition of H3K36me3 increases. The prediction from this model that the same genomic regions or nucleosomes that are marked by H3K23me3 during initiation become marked by H3K36me3 remains to be tested. Both during initiation and maintenance of TEI, in every individual worm, some germ cells escape the maintenance of the altered H3K27me3 epialleles and reconstruct wild-type H3K27me3 patterns. This competition between the replicative TEI and the reconstructive TEI results in a partial penetrance of the inherited fertility defects even within individual worms.

Genomic domains enriched in H3K27me3 and H3K36me3 are mutually exclusive in several model organisms (Kaneshiro et al, 2022; Schmitges et al, 2011; Dorighi and Tamkun, 2013). We found that the switch from H3K27me3 domains to H3K36me3 domains required the transient deposition of H3K23me3 by SET-32. Maybe because of the transient nature of H3K23me3, point mutations at H3K23 or loss-of-function mutations of H3K23me3 writer or reader proteins are not associated with any obvious defects (Zhebrun et al, 2025), and mutants of *set-32* are viable (Woodhouse et al, 2018; Andersen and Horvitz, 2007; Kalinava et al, 2017). SET-21 has been described as the second H3K23me3 HTM that acts in parallel to SET-32 (Zhebrun et al, 2025). SET-21 appears to be most expressed during diakinesis and contributes less to the overall H3K23me3 levels than SET-32 (Zhebrun et al, 2025), and we therefore did not test it in our transgenerational assay. SET-32 was previously identified in epigenetic suppressor screens (Greer et al, 2014; Okabe et al, 2021; Wan et al, 2021). It was found to be required for the initiation of heritable epigenetic phenotypes such as an increased lifespan caused by the reduction of H3K4me3 levels or the silencing of a GFP reporter transgene (Woodhouse et al, 2018; Kalinava et al, 2018; Ashe et al, 2012; Greer et al, 2014). In line with these previous studies, we found that removal of SET-32 does not have a suppressive effect on *H3.3K27M*^tet^-expressing P0, but that SET-32 is required for the initiation of replicative EI during the transition from P0 to F1. We propose that H3K23me3 is a transitory histone PTM required to initiate a heritable epigenetic state in response to induced changes in the epigenome. We can only speculate what mediates the transition from H3K23me3 to H3K36me3. Methylation at position K23 may be incompatible with additional methylation at position K36. If this is the case, MES-4-mediated deposition of H3K36me3 would require either the activity of a yet to be identified demethylase, or a replication-dependent nucleosome turnover during germline development of the subsequent generation.

To experimentally select subfertile animals in each generation, we used an arbitrary cutoff of <50 offspring, resulting in an observation of 30–40% replicative TEI and 60–70% reconstructive TEI in each generation (Fig. 2B). Similarly, for quantifying germ cells with altered H3K27me3 landscapes, we only scored those as “altered” that resembled cells expressing H3.3K27M in the P0 (Fig. 2F). However, we observed a spectrum for both phenotypes, with a broad range of embryos laid (Fig. 2C), and intermediate stages of reconstruction of the wildtype H3K27me3 landscape (Fig. 2F). We therefore likely underestimate the penetrance of the TEI. However, our results resemble the low penetrance of epigenetically inherited phenotypes observed in previous studies (Barucci et al, 2020; Demoinet and Roy, 2018; Greer et al, 2016, 2014, 2011; Heestand et al, 2018; Houri-Zeevi et al, 2021; Kalinava et al, 2018, 2017; Kaneshiro et al, 2022; Klosin et al, 2017; Lee et al, 2019; Lev et al, 2017; Posner et al, 2019; Toker et al, 2022; Woodhouse et al, 2018). The spectrum of germ cells with either replicated or reconstructed H3K27me3 distributions is observed within individuals (Fig. 1E), and not just within the population, explaining why in every generation, individual worms can give rise to a mix of fertile and subfertile offspring. We have previously shown that the global loss of H3K27me3 causes gene expression changes at many genes, but that de-silencing of the *kgb-1* gene is mainly responsible for the reduction in brood size (Delaney et al, 2019). The observed partial reconstruction of H3K27me3 domains in some germ cells (Fig. 2F) can therefore explain the imperfect correlation between chromatin defects and reduced brood sizes, as the latter depends on whether the *kgb-1* locus is targeted by the partial reconstruction. How specific regions are targeted by PRC2 for H3K27me3 reconstruction is not well understood, and the amount of PRC2 is likely limiting this process. Similar observations were made for sperm-inherited epialleles lacking H3K27me3, where the majority of loci regained H3K27me3 in the offspring, while few regions could not reconstruct H3K27me3, and instead maintained H3K36me3 marks (Kaneshiro et al, 2022, 2019). These observations support the concept that the heritability of altered H3K27me3 and H3K36me3 chromatin landscapes is subject to the continuous competition between PRC2 and MES-4.

PRC2 is essential for development, as homozygous mutations in PRC2 result in defective lineage specification and body patterning in flies and mammals (Grijzenhout et al, 2016; Lewis, 1978; O’Carroll et al, 2001; Pasini et al, 2004). Additionally, several monoallelic, recessive loss-of-function mutations in PRC2 (likely reducing the overall HMT activity) are associated with developmental disorders such as Weaver Syndrome, suggesting that not only the zygotic presence, but also the post-embryonic maintenance of H3K27me3 epialleles is important in humans (Lui et al, 2018; Imagawa et al, 2017). Therefore, defining the mechanisms underlying the maintenance of H3K27me3 is crucial for understanding cellular epigenetic memory in health and disease. In *C. elegans*, mutations in PRC2 result in a Mes phenotype due to the failure in the maintenance of silencing X-linked genes in the germ line (Gaydos et al, 2012; Bender et al, 2006). This Mes phenotype can be interpreted as an epigenetic phenotype, as loss of maternal PRC2 only affects the grandchildren, but the sterility prevents the analysis of future generations (Gaydos et al, 2014). Here, we take advantage of the inhibition of PRC2 by the transient expression of H3.3K27M (Delaney et al, 2019) to demonstrate that EI can persist beyond the following generation. This approach resembles the transient inhibition of PRC2 recently described in *Drosophila*, which leads to an irreversible derepression of genes linked to tumorigenesis (Parreno et al, 2024). Our results show that such a derepressed state can be heritable and under selection extend for many generations in *C. elegans*.

The balance between reconstructive and replicative TEI by opposing histone PTMs in the germ line appears to be a general principle for heritable gene expression states, and may provide plasticity and epigenetic variation across generations (Jablonka, 2013; Patel and Wang, 2013). Here, we have shown the long-term inheritance of an altered H3K27me3 landscape, which is maintained by a transient spreading of H3K23me3 followed by H3K36me3. We propose that this inheritance of epigenome-based phenotypes can provide a mutation-independent mechanism for adaptation to the environment.

There are several limitations to our study. It investigates transgenerational epigenetic inheritance and probes the epigenome at every generation in a specific developmental window of the germline. This allows us to determine what changes from one generation to the next, but does not allow us to follow the dynamic processes that occur during development. The stochastic nature of the epigenetic inheritance and the relatively low penetrance of the phenotypes prevent us from generating large epigenetically homogenous populations that would be required for omics analyses in later generations, e.g., to probe if the same nucleosomes or genomic regions are marked by H3K23me3 during establishment and by H3K36me3 during maintenance. Detailed epigenome profiling of individual germ cells is currently hampered by the technical limitations to obtaining completely resolved single-cell epigenomes for *C. elegans*.

## Methods

**Table:** Reagents and tools table

Reagent/resource	Reference or source	Identifier or catalog number
**Experimental models**		
N2 (*C. elegans*)	CGC	
FAS158 (*C. elegans*)	Steiner lab	
**Recombinant DNA**		
TC374	Mao et al, 2019	Tetracyclin Array
pIO1	Steiner lab	Tetracyclin Array
pIO9	Steiner lab	RNAi
pIO47	Steiner lab	RNAi
**Antibodies**		
Rat anti-OLLAS	Novus Biologicals	NBP1-06713
Mouse anti-H3K27me2/3	Active motif	39535
Rabbit anti-H3K23me3	Active motif	61499
Rabbit anti-H3K36me3	Abcam	ab9050
anti-H3 antibody	Abcam	Ab1791
Anti-Rat Cy3	Jackson ImmunoResearch	712-165-150
Anti-Mouse Cy5	Jackson ImmunoResearch	715-175-150
Anti-Rabbit Alexa488	Thermo Fisher Scientific	A-11008
Anti-Rat HRP-coupled secondary	Cell Signaling	7077
Anti-Rabbit HRP-coupled secondary	Cell Signaling	7074
**Oligonucleotides and other sequence-based reagents**		
**Chemicals, enzymes and other reagents**		
poly-L-lysine	Sigma-Aldrich	P8920-100ML
VECTASHIELD Antifade Mounting Medium	Sigma-Aldrich	H-1200
Clarity Western ECL Substrate	Bio-Rad	1705061
DAPI	Roche	1023627600
TRIZOL	Invitrogen	15596026
IPTG	AppliChem	A1008
Tetracycline hydrochloride	Sigma-Aldrich	T7660-5G
Bromophenol Blue	Fisher Bioreagents	BP115-25
SDS	Serva Electrophoresis	20765.02
2-mercaptoethanol	Thermo Fisher Scientific	125472500
Trizma® base	Sigma-Aldrich	T1503-1KG
Tween-20	Sigma-Aldrich	P9416-100ML
**Software**		
Fiji	Schindelin et al, 2012	
MMMEA macro	Schindelin et al, 2012	
EZcolocalization	Stauffer et al, 2018	
R v4.5.3	The R Project for Statistical Computing	
Salmon v1.20.3	Patro et al, 2017	
Tximport v1.34.0	Soneson et al 2015	
DESeq2 v1.46.0	Love et al, 2014	
Tidyverse v2.0.0	Wickham et al, 2019	
Affinity Designer	Affinity Studio	
**Other**		
Illumina NovaSeq 6000	Illumina	
Leica LSM800	Leica	
Amersham ImageQuant 800	Amersham	

### *C. elegans* strains and culture

*C. elegans* strains were grown using standard conditions for maintenance at 20 °C (Brenner, 1974). All experiments were performed at 25 °C unless otherwise stated. Animals were maintained on regular NGM (0.25% peptone, 0.3% NaCl, 1.5% agar, 1 mM CaCl_2_, 1 mM MgSO_4_, 25 mM KH_2_PO_4_, pH 6.0, 25 mM K_2_HPO_4_, pH 6.0, and 5 µg/mL cholesterol) seeded with *E. coli* OP50 or HT115. N2 (Bristol strain) was used as the wildtype. The generation of tetracycline-inducible transgenes expressing H3.3K27M was based on the sequence of the previously published *his-72(uge72)* allele containing a C-terminal OLLAS tag (Delaney et al, 2019). The transgene (referred to as *H3.3K27M*^tet^) was expressed from an extrachromosomal array obtained by microinjection of plasmids TC374 (containing the transgenic tetracycline-inducible driver) (Mao et al, 2019) and pIO1 (containing the *his-72K27M::OLLAS* responder transgene) into N2 (Fig. 1B). This resulted in the strain FAS158(*ugeEx108*[rpl-28p::rTetR::QF::P2A::mKate::T2A::TetR::pie-1 + 7xTetO::pes-10p::his-72[K27M]::ollas his-72 3’UTR]), which was used for all experiments. *H3.3K27M*^tet^ induction was done on 1 ng/µl tetracycline-containing NGM plates seeded with HT115 unless otherwise indicated.

### Transgenerational subfertility assay

The approach for initiating fertility defects and following their inheritance is described in (Fig. 2A). In all experiments that assess fertility, we singled worms at larval stages before any germline or fertility phenotypes were visible to avoid observer bias. About 10–12 P-1 animals (parents of P0 generation) were selected based on the presence or absence of the extrachromosomal *H3.3K27M*^tet^ array and placed at 25 °C when they were at the L3-L4 larval stage. From these P-1 plates, >50 L3/early L4 animals (before any phenotypes were apparent) of the P0 generation were singled. *P0*^array^ animals contained the H3.3K27M^tet^ array, while *P0*^wt^ animals were derived from wildtype P-1 animals and thus did not contain the array. After 3–4 days, the brood sizes of these P0 animals were scored under a dissecting microscope (Leica S9E stereo microscope) and categorized into fertile and subfertile. Fertile animals lay more than 50 viable embryos, while subfertile worms lay less than 50 viable embryos. For the analysis of the inheritance, only individuals without the H3.3K27M^tet^ array were selected, so that all worms in the *F* generations were genetically wildtype. For the *F1* generation, >50 offspring of subfertile animals from the *P0*^array^ were randomly selected at the L3/L4 stage and singled for the analysis of phenotypes in the *F1*^subfertile^ generation. As control, >50 offspring of fertile animals from the *P0*^wt^ were randomly selected at the L3/L4 stage and singled for the analysis of phenotypes in the *F1*^wt^ generation. This procedure was repeated for subsequent *F* generations, with the selection and analysis of >50 offspring of subfertile* F*^subfertile^ animals and >50 offspring of fertile* F*^wt^ animals in each generation. Fertility level comparisons were performed in three biological replicates. Statistical tests applied for each experiment were specified in the corresponding figure legends.

### RNAi treatment and temperature shift assay

The standard RNAi by feeding protocol (Kamath et al, 2001) was used. Briefly, overnight-grown RNAi precultures in LB medium containing ampicillin at 37 °C were diluted 10x in the same medium and incubated for 3–4 h at 37 °C (until OD values reached up to 0.6–0.8), followed by 4 mM IPTG induction for another 4 h at 37 °C. The cultures were concentrated by centrifugation before seeding onto freshly prepared RNAi NGM (±1 ng/µl tetracycline, 1 mM IPTG, and 25 µg/ml carbenicillin). *mes-4(RNAi) and set-32(RNAi)* clones pIO47 and pIO9 were generated by Gibson assembly using L4440 as a backbone (Gibson et al, 2009).

For *mes-4(RNAi*), L3/L4 from the P0, F1, F2, F5, F6, and F7 generations were exposed to RNAi by feeding, and 40–50 offspring were singled on individual plates for fertility assessment. Only the first generation following *mes-4(RNAi)* could be assessed, as the second generation showed the expected Mes phenotype. For *set-32(RNAi*), worms were exposed to RNAi by feeding from P0-F2, F1-F2, and F5-F8. For *kgb-1(RNAi)*, worms were exposed to RNAi by feeding from P0-F1, F1-F2, F3-F4, and F4-F5. As a control for the RNAi treatment, worms were continuously exposed to bacteria containing the empty L4440 RNAi vector. Temperature shift experiments were done as RNAi experiments, except that plates were shifted from 25 °C to 20 °C from P0-F1, F1-F2, F3-F4, and F4-F5.

### Immunofluorescence staining and image analysis

Worm gonads were dissected in 4 µl of anesthetizing DB buffer (1.6% sucrose, 73 mM HEPES, pH 6.9, 40 mM NaCl, 5 mM KCl, 2 mM MgCl_2_, 10 mM EGTA, pH 7.5, 0.1% NaN_3_) on ten-well microscope slides (Thermo Fisher Scientific) with a 21 G needle (BD Microlance). Dissected gonads were transferred to poly-L-lysine-coated (0.1% (w/v); Sigma-Aldrich) three-square-well microscope slides (Thermo Fisher Scientific). Slides were placed on a dry-ice cold metal plate for at least 30 min. Samples were fixed in methanol for 10 min. The fixatives were stored at −20 °C and reused for a maximum of ten experiments. Slides were washed three times in PBS for 5 min and incubated with 1:300 anti-OLLAS (Novus Biologicals NBP1-06713), 1:300 anti-H3K27me2/3 (Active motif 39535), 1:600 anti-H3K23me3 (Active motif 61499) or 1:2000 anti-H3K36me3 (Abcam ab9050) antibodies overnight at 4 °C in a humid chamber. Samples were washed in PBS-Tween-20 three times for 5 min and incubated with 1:1000 times diluted Cy3- (Jackson ImmunoResearch) or Alexa488 (Thermo Fisher Scientific) secondary antibodies for 1.5 h at room temperature in the dark. Samples were washed with PBS-Tween-20 three times for 5 min, stained with 2 µg/ml DAPI for 15 min, and mounted with VECTASHIELD Antifade Mounting Medium (H-1200). 2D and confocal images were obtained using a Leica DM5000B with 63x oil objectives (NA = 1.25) and a Leica LSM800 confocal microscope with 63x oil objectives (NA = 1.40), respectively. Representative confocal images show maximum projections of 0.1-μm Z-sections of the entire pachytene nuclei.

For quantifying the extent of co-localization of histone marks on the chromatin of stained pachytene nuclei, MMMEA macro and EZcolocalization plugin in Fiji were used (Stauffer et al, 2018; Schindelin et al, 2012). The maximum Z projections for each nucleus were stacked based on the DAPI signal. Graphs were generated based on the user-defined parameters and the obtained measurements using a custom R script. The percentage of pachytene cells showing altered H3K27me3 patterns per gonad were scored manually using Fiji (Schindelin et al, 2012).

### Western blotting

Western blotting was carried out according to standard procedures. For detecting H3.3K27M expression, 40 worms per condition were picked into 20 µL of M9 buffer, and an equal volume of 2X SDS-PAGE Loading Buffer was added. Samples were sonicated using a Bioruptor (Eppendorf metallic stand) at high power for ten cycles of 30 s ON/30 s OFF at 4 °C. Lysates were then boiled for 3 min and 20 µL were used for Western blot. OLLAS-tagged H3.3K27M was detected with an anti-OLLAS antibody (Novus Biologicals NBP1-06713), and H3 (loading control) was detected using an anti-H3 antibody (Abcam Ab1791). HRP-coupled secondary antibodies (Rat: Cell Signaling #7077, Rabbit: Cell Signaling #7074) were revealed by ECL (Bio-Rad #1705061) using an Amersham ImageQuant 800.

### RNA-seq and expression analysis

For the RNA-seq analysis, we collected germ lines from *P0*^wt^ (wildtype control; *n* = 3), subfertile individuals from *P0*^array^ (*n* = 2), subfertile individuals from *F3*^subfertile^ (*n* = 2), and fertile individuals from *F3*^subfertile^ (*n* = 2). We estimated whether individual animals were fertile or subfertile by letting them lay eggs until the second day of adulthood, and then dissected germ lines for RNA extraction. Worms were washed in PBS and transferred to PBS supplemented with 0.1% sodium azide and 0.01% Tween-20. Gonads were extruded and severed from the carcass by cutting worms with a 26-gauge needle. Individual gonads were transferred using glass needles into 10 µl of TRIzol (kept on ice) in a three-well glass block, and dissolution of the gonad was confirmed visually under the microscope. Approximately 20 gonads were collected per sample. The TRIzol volume was adjusted to 100 µl, and RNA was extracted according to the manufacturer’s protocol.

RNA-seq libraries were prepared by the iGE3 Genomics Platform (University of Geneva) using the Illumina Stranded mRNA protocol with polyA selection, and sequenced with the 50nt single-end protocol on a NovaSeq 6000. Single-end sequencing reads were quasi-mapped to the *C. elegans* transcriptome (WBcel235) using Salmon v1.10.3 (Patro et al, 2017) with GC bias correction and mapping validation. A decoy-aware index was constructed using the full WBcel235 genome sequence as a decoy. Transcript-level quantifications were imported into R and summarized to gene level using tximport v1.34.0 (Soneson et al, 2015). Differential gene expression analysis was performed using DESeq2 v1.46.0 (Love et al, 2014). Genes were considered differentially expressed at a Fold Change of ≥2 and an FDR of ≤0.01.

### Data representation and statistical analysis

All data analysis, statistical significance testing, and plot rendering were performed in R using tidyverse v2.0.0 (Wickham et al, 2019). The figures were assembled in Affinity Designer and Inkscape. Quantification of brood sizes, fertility, nuclei per gonad, or chromatin occupancy are derived from biological replicates.

## Supplementary information

Peer Review File

Expanded View Figures

## Data Availability

Raw data underlying the figures is accessible at YARETA using the link: https://doi.org/10/hb9nnd. Sequencing data is available on NCBI Gene Expression Omnibus under accession number GSE337109: https://ncbi.nlm.nih.gov/geo/query/acc.cgi. The source data of this paper are collected in the following database record: biostudies:S-SCDT-10_1038-S44318-026-00907-9.
