# Supplementary material for: Transgenerational inheritance of altered H3K27me3 in wild-type Caenorhabditis elegans
Source: EMBO J. 2026 Sep 18;45(20):7226–45. doi: 10.1038/s44318-026-00907-9 (PMC13672289; doi:10.1038/s44318-026-00907-9)
Supplement: Supplementary file 1 [file 44318_2026_907_MOESM1_ESM.pdf]

THE EMBO JOURNAL

Transgenerational inheritance of altered H3K27me3 in wild-type Caenorhabditis elegans

İsa Özdemir, François-Xavier Stubbe, Kamila Delaney, Joanna Wenda, and Florian Steiner

Corresponding author(s): Florian Steiner (florian.steiner@unige.ch)

Review Timeline:

Submission Date:	20th Nov 25
Editorial Decision:	22nd Dec 25
Revision Received:	7th Jul 26
Editorial Decision:	3rd Aug 26
Revision Received:	10th Aug 26
Accepted:	25th Aug 26

Editor: Hartmut Vodermaier

Transaction Report:

(Note: With the exception of the correction of typographical or spelling errors that could be a source of ambiguity, letters and reports are not edited. Depending on transfer agreements, referee reports obtained elsewhere may or may not be included in this compilation. Referee reports are anonymous unless the Referee chooses to sign their reports.)

Prof. Florian Steiner
University of Geneva
Department of Molecular and Cellular Biology
Switzerland

22nd Dec 2025

Re: EMBOJ-2025-123096
Transgenerational inheritance of altered H3K27me3 in wildtype Caenorhabditis elegans

Dear Florian,

Thank you for submitting your manuscript on transgenerational H3K27me3 inheritance for our consideration. I sent it to three expert referees, who have now returned the reports that you will find copied below. As you will see, all referees appreciate your approaches and experimental strategy for addressing this important question, and would in principle support publication of an adequately revised version. However, they raise several important issues of both experimental and conceptual nature that would need to be satisfactorily addressed prior to acceptance. I will not repeat the more technical points here, but key conceptual questions regard the mechanistic understanding of the proposed "chromatin handoff", and the current limitation to IF studies and lack of higher-resolution information.

Faced with these critical assessments, I would like to give you a chance to respond to the referees' concern by way of a revised manuscript, but I agree with referee 3 that this would require at least some further insights into underlying mechanisms of transgenerational inheritance of histone marks and fertility defects and their connections. Since it is not clear if and how this might be addressed during a single round of regular revision allowed by our editorial policies, I would encourage you to contact me with a revision proposal and preliminary point-by-point response already during the early stages of your revision work. Depending on follow-up discussions of these plans, we could also explore whether a less completely revised manuscript might alternatively become suitable for publication in one of our sister journals like EMBO Reports or Life Science Alliance. I would also be happy to extend the revision time beyond the regular three months if this should be needed. Our 'scooping protection' (meaning that competing work appearing elsewhere in the meantime will not affect our considerations of your study) would of course remain valid also throughout such an extension.

Further information on preparing, formatting and uploading a revised manuscript can be found below and in our Guide to Authors. Thank you again for the opportunity to consider this work for The EMBO Journal, and I look forward to hearing from you soon.

With kind regards,

Hartmut

Hartmut Vodermaier, PhD
Senior Editor, The EMBO Journal
h.vodermaier@embojournal.org

*** PLEASE NOTE: All revised manuscript are subject to initial checks for completeness and adherence to our formatting guidelines. Revisions may be returned to the authors and delayed in their editorial re-evaluation if they fail to comply to the following requirements. As a first step please read our guidelines for revised submissions:
https://link.springer.com/journal/44318/submission-guidelines#cms-Revised-submissions

1) Every manuscript requires a Data Availability section (even if only stating that no deposited datasets are included). Primary datasets or computer code produced in the current study have to be deposited in appropriate public repositories prior to resubmission, and reviewer access details provided in case that public access is not yet allowed.

2) Each figure legend must specify
- size of the scale bars that are mandatory for all micrograph panels
- the statistical test used to generate error bars and P-values
- the type error bars (e.g., S.E.M., S.D.)
- the number (n) and nature (biological or technical replicate) of independent experiments underlying each data point
- Figures may not include error bars for experiments with n<3; scatter plots showing individual data points should be used instead.

3) Revised manuscript text (including main tables, and figure legends for main and EV figures) has to be submitted as editable text file (e.g., .docx format). We encourage highlighting of changes (e.g., via text color) for the referees' reference.

4) Each main and each Expanded View (EV) figure should be uploaded as individual production-quality files (preferably in .eps, .tif, .jpg formats). For suggestions on figure preparation/layout, please refer to our Figure Preparation Guidelines: https://media.springernature.com/original/springer-cms/rest/v1/content/27825798/data/v1

5) Point-by-point response letters should include the original referee comments in full together with your detailed responses to them (and to specific editor requests if applicable), and also be uploaded as editable (e.g., .docx) text files.

6) Please complete our Author Checklist, and make sure that information entered into the checklist is also reflected in the manuscript; the checklist will be available to readers as part of the Review Process File.

7) All authors listed as (co-)corresponding need to deposit, in their respective author profiles in our submission system, a unique ORCiD identifier linked to their name. Please see our Guide to Authors for detailed instructions.

8) Please note that supplementary information at EMBO Press has been superseded by the 'Expanded View' for inclusion of additional figures, tables, movies or datasets; with up to five EV Figures being typeset and directly accessible in the HTML version of the article.

9) To facilitate reproducibilty and cross-laboratory adoption of methodologies, please structure the Materials & Methods section as outlined in our guide to authors, including a completed Reagents and Tools Table.

10) Digital image enhancement is acceptable practice, as long as it accurately represents the original data and conforms to community standards. If a figure has been subjected to significant electronic manipulation, this must be clearly noted in the figure legend and/or the 'Materials and Methods' section. The editors reserve the right to request original versions of figures and the original images that were used to assemble the figure. Finally, we generally encourage uploading of numerical as well as gel/blot image source data.

At EMBO Press we ask authors to provide source data for the main manuscript figures. You will receive a separate email with instructions for providing source data with your revised manuscript, including how to upload and organize the files.

In the interest of ensuring the conceptual advance provided by the work, we recommend submitting a revision within 3 months (22nd Mar 2026). Please discuss the revision progress ahead of this time with the editor if you require more time to complete the revisions. Use the link below to submit your revision:
Link Not Available

If you choose to alternatively have this study further considered by another EMBO Press publication, please use the following hyperlink to directly transfer the manuscript, optionally with inclusion of referee reports and identities:
Link Not Available

Referee #1:

In this manuscript, the authors describe a multigenerational, selectable subfertility phenotype associated with aberrant redistribution of H3K27me3 in the germline. They propose a two-step mechanism in which an early wave of H3K23me3 deposition is required to establish ectopic H3K36me3, which in turn antagonizes normal H3K27me3 redistribution and stabilizes the inherited chromatin state across generations.

The work addresses an important and timely question: how distinct histone modifications can be sequentially rewired to generate a metastable, inherited chromatin configuration with phenotypic consequences. The genetic logic is strong, the generational analyses are carefully executed, and the study provides an intriguing conceptual framework separating initiation and maintenance phases of epigenetic inheritance.

At the same time, several key mechanistic claims would benefit from clarification, additional controls, or more cautious wording. In particular, the reliance on IF-based chromatin readouts, the temperature regime used throughout, and the interpretation of H3K23me3 and H3K36me3 dynamics raise important questions that should be addressed to strengthen the conclusions.

Major points
1. Temperature dependence and generality of the inheritance mechanism
Most experiments are performed at 25{degree sign}C, a condition that is itself stressful for C. elegans fertility. While this choice is

experimentally effective, it remains unclear whether the observed chromatin redistribution and inheritance reflect a general epigenetic mechanism or a temperature-enabled regime.
Specifically, is the multigenerational inheritance (penetrance, stability, reversibility) maintained at lower temperature, or does it require sustained growth at 25{degree sign}C? Even limited experiments at 20{degree sign}C, or temperature-shift assays after the phenotype is established, would greatly clarify the physiological relevance and robustness of the mechanism. For example, it would be informative to determine whether shifting the inheriting generations to 20{degree sign}C suppresses or attenuates the phenotype.

2. Specificity of SET-32 as the H3K23me3 methyltransferase.
The model assigns a central initiating role to SET-32-dependent H3K23me3 deposition. While the genetic requirement for set-32 is convincing, the statement that SET-32 is the only germline H3K23me3 methyltransferase is not supported by current literature.
This conclusion is primarily based on loss of IF signal in set-32 mutants, whereas a recent study (Zhebrun et al., Genetics 2025) identified SET-21 as an additional germline-expressed H3K23 methyltransferase. The authors should revise their wording to reflect SET-32 as a major or dominant enzyme rather than the sole H3K23me3 HMT. Ideally, the authors should test whether SET-21 contributes to initiation or modulates penetrance of the inherited phenotype, or explicitly discuss why SET-21 is unlikely to participate in this paradigm.

3. Relationship between early H3K23me3 and later H3K36me3 domains.
A central claim is that early H3K23me3 deposition enables later accumulation of ectopic H3K36me3, which maintains the inherited state. However, it remains unclear whether the same chromatin regions are involved in this temporal transition.
Specifically:
Are regions initially marked by H3K23me3 the ones that later acquire H3K36me3?
Or are these changes correlated at the nuclear level but not spatially coincident?
Given the reliance on IF-based analyses, additional spatial quantification (e.g. higher-resolution imaging or nucleus-level colocalization metrics) would strengthen the argument for a true chromatin "handoff" rather than parallel global changes.

4. Conceptual relationship between repressive and active-associated histone marks.
H3K23me3 is generally associated with repressive chromatin, whereas H3K36me3 correlates with transcriptionally active gene bodies. How a repressive mark gives rise to, or is replaced by, an active-associated mark remains mechanistically unresolved.
Key questions include:
Is ectopic H3K36me3 driven by transcription in later generations?
Is MES-4 acting in a transcription-independent manner at atypical chromatin domains?
Are transcriptional changes detectable that correlate with the maintenance phase and fertility defects?
While the authors acknowledge the limitations of IF-based profiling, establishing some conceptual or experimental link between chromatin state and transcriptional output would considerably strengthen the mechanistic framework.

5. Role of kgb-1 in the inherited phenotype.
The authors show that suppression of kgb-1 partially rescues the P0 fertility phenotype, implicating stress-response pathways in the initiation phase. However, it remains unclear whether kgb-1 also plays a role in the inherited phenotype.
Does kgb-1 contribute to subfertility or chromatin defects in inheriting generations, or is its role restricted to the initiating P0 context? Testing kgb-1 suppression in F1/F2 inheriting lineages would clarify whether the same effector pathways operate during initiation and maintenance.

Minor points
- Reporting IF variability and nucleus-to-nucleus heterogeneity more explicitly would help interpret penetrance.
- Some statements in the Discussion could be phrased more cautiously, given the absence of genome-wide chromatin or transcriptional profiling.

Overall recommendation
This is a conceptually interesting and carefully executed study that would be suitable for EMBO Journal provided the authors address the mechanistic and generality issues outlined above. The work has the potential to make a significant contribution to our understanding of multistep chromatin-based inheritance, but several key conclusions would benefit from clarification and additional support.

Referee #2:

The submission by Ozdemir et al. investigated whether an altered distribution of the critical histone mark H3K27me3 generated in the parental generation is transmitted to offspring. They used C. elegans as their model system and achieved alteration of the distribution of H3K27me3 by expressing H3.3K27M in the parental generation. Previous studies had shown that expression of H3.3K27M 1) causes H3K27me3 to become less concentrated on the autosomes while it persists on the X chromosomes in the germline and 2) results in endomitotic oocytes and reduced fertility. The authors used a tetracycline-inducible transgene to express H3.3K27M in the parental generation and dialed the tetracycline level to allow worms to have adequate fertility to

analyze their offspring in subsequent generations. The transgene also expressed a fluorescent marker, to allow the authors to select offspring that had lost the transgene, so that they could follow the inheritance of altered H3K27me3 without ongoing expression of H3K27M.

The authors analyzed epigenetic inheritance of an altered pattern of H3K27me3 in offspring 1) at the single-cell level by using immunofluorescence to image in individual cells whether the distribution of H3K27me3 remained altered or reverted to wild type and 2) at the whole-organism level by measuring brood size. Based on those two levels of analysis, the major finding is that an altered pattern of H3K27me3 induced in the parental generation and associated reduced fertility are passed to a significant percentage of the offspring for at least 15 generations. Not surprisingly, there is cell-to-cell and organism-to-organism variation. The results support epigenetic transmission of an altered H3K27me3 distribution to some offspring and restoration of a close-to-wild-type H3K27me3 distribution in other offspring.

To investigate initiation and maintenance of an altered H3K27me3 distribution, the authors focused on H3K23me and H3K36me. Both marks displayed more widespread distributions in samples with reduced H3K27me3, and RNAi knock-down of the enzymes that generate each mark influenced the distribution of H3K27me3 and fertility. Based on the generations in which those effects were seen, the authors conclude that H3K23me is important for the initiation phase, and H3K36me is important for the maintenance phase.

All in all, this study uses a clever approach to document the transmission of altered histone marking in one generation to future generations, and to identify key regulators of that transmission.

One limitation of the study (acknowledged by the authors) is the absence of high-resolution information (e.g. from ChIP-seq) on the genome-wide distribution of H3K27me3. But most high-resolution approaches require populations of cells/nuclei/animals and therefore present an average, which would not capture the cell-to-cell variability seen by immunofluorescence and the worm-to-worm variability seen by fertility tests.

Comments:
1) The quality of the documentation of altered distributions of H3K27me3 is variable. The images in Figure 1D are too dark and fuzzy to discern the different staining patterns. Figure 2D is better. Supplemental Figure S1B is good and furthermore illustrates the range of altered patterns and points out in the legend that only complete replicative EI was considered as "altered", whereas full and partial reconstructive EI was considered as "wildtype", as mentioned in lines 223-225 and 393-395. I think that Figure S1B should be moved into the main paper. Additionally, the authors should consider showing that staining in black and white in addition to the merged colors, as red and blue are hard to tease apart in merged images.

2) The Tet strategy needs a bit more explanation. According to the Mao et al. 2019 reference, it looks like ACTIVATOR is reverse Tet REPRESSOR fused to VP16. Tetracycline exposure causes the ACTIVATOR to bind the TRE - why doesn't the REPRESSOR still bind as well?

3) Lines 167-168 and Figures 1C and 2A differ in whether both P-1 and P0 express H3.3K27M, or only P0. Please clarify.

4) I was confused about generations in Figure 2C and 2E and the associated text in Results. Lines 217-218 say "We determined whether individual P0wt/Fwt or P0array/Fsubfertile animals were fertile or subfertile", which doesn't make sense. In Figure 2E, does the label Fsubfertile refer to the parent of the data points, since the data points show both fertile (triangles) and subfertile (dots). Same question for Figure 2C. Please clarify in the figure and text.

5) In Figure 4C, should the generations at the bottom be P0, F1, F2 instead of F1, F2, F3?

Line 188 - fix "sub-phenotyes"
Line 206 - should Figure 1D be cited, instead of Figure 1C?
Line 531 - delete "chromodomain proteins"

Referee #3:

In this manuscript, the authors investigate transgenerational epigenetic inheritance of chromatin defects due to the effect of transient expression of an H3.3K27M. This has been shown before to lead to genome-wide chromatin changes in C. elegans but what the authors do now is to use this system to study the inheritance of the disordered H3K27me3 that results from H3.3K27M overexpression over several generations. This is because they can link phenotype (brood size) to H3K27me3 levels (via IF) at the single individual level, essential as the inheritance of the disordered chromatin is somewhat stochastic.
Overall, within the framework of the experiments performed the results are solid, with appropriate statistics and controls, and convincing. The weakness, however, is that the authors cannot provide a clear molecular link between the fertility defects and the 'disordered' landscape because IF is such a blunt tool, which cannot identify the specific genes, or set of genes, that are

responsible. This is of fundamental importance because the model that the authors propose is that there is stochastic, imperfect, inheritance of the defective landscape- this implies, though, that even individual worms with disordered H3K27me3 as measured by IF, might actually differ in which regions of the genome are affected. It's obviously very hard to deal with this variability at the chromatin level, because a genome-wide ChIP-seq is impractical from single worms. However, there are some approaches that could be taken:
i) could pools of worms assayed for low fertility be used for ChIP-seq experiments? For example, many single worms could be plated, allowed to lay eggs for a fixed period, and then frozen in an individual tube. After analysing how many eggs hatched (brood size) the worms in the different tubes could be pooled into either high or low fertility groups and ChIP done on them. I think that 100 worms for each group (so possibly 200 total) would be enough. This would be a considerable effort, but I think would be possible.
ii) Single worm RNAseq has been developed (see for example https://www.biorxiv.org/content/10.1101/2025.11.01.686022v1 or https://elifesciences.org/articles/106525). This could be combined with fertility assay (as above) and each worm studied for its RNA profile. The key question would be whether there are predictable gene expression correlates associated with fertility defects, ie that most worms showing the H3K27M induced defects will have changes in expression of certain genes. These could be linked to chromatin domains according to known maps of WT chromatin, to see whether (say) upregulated genes are enriched for H3K27M domains.
I feel that, for a journal such as EMBO, something to bolster the molecular chain of causation between defective H3K27me3 and fertility and transgenerational epigenetic inheritance would be important, so perhaps the authors could think about these suggestions, or alternatively think of something else that might address this key point.

Response to Reviewers

Referee #1:

In this manuscript, the authors describe a multigenerational, selectable subfertility phenotype associated with aberrant redistribution of H3K27me3 in the germline. They propose a two-step mechanism in which an early wave of H3K23me3 deposition is required to establish ectopic H3K36me3, which in turn antagonizes normal H3K27me3 redistribution and stabilizes the inherited chromatin state across generations.

The work addresses an important and timely question: how distinct histone modifications can be sequentially rewired to generate a metastable, inherited chromatin configuration with phenotypic consequences. The genetic logic is strong, the generational analyses are carefully executed, and the study provides an intriguing conceptual framework separating initiation and maintenance phases of epigenetic inheritance.

At the same time, several key mechanistic claims would benefit from clarification, additional controls, or more cautious wording. In particular, the reliance on IF-based chromatin readouts, the temperature regime used throughout, and the interpretation of H3K23me3 and H3K36me3 dynamics raise important questions that should be addressed to strengthen the conclusions.
We thank the reviewer for the thoughtful comments and suggestions.

Major points
1. Temperature dependence and generality of the inheritance mechanism
Most experiments are performed at 25{degree sign}C, a condition that is itself stressful for C. elegans fertility. While this choice is experimentally effective, it remains unclear whether the observed chromatin redistribution and inheritance reflect a general epigenetic mechanism or a temperature-enabled regime.
Specifically, is the multigenerational inheritance (penetrance, stability, reversibility) maintained at lower temperature, or does it require sustained growth at 25{degree sign}C? Even limited experiments at 20{degree sign}C, or temperature-shift assays after the phenotype is established, would greatly clarify the physiological relevance and robustness of the mechanism. For example, it would be informative to determine whether shifting the inheriting generations to 20{degree sign}C suppresses or attenuates the phenotype.

We have tested if shifting from 25°C to 20°C at generations P0-F1, F1-F2, F3-F4 and F4-F5 can suppress the fertility defects. We found that shifting to 20°C at any generation suppressed the fertility defects in the following generation, as shown in new Figure 3B.

This result is consistent with our previous study (Delaney et al 2019), where we showed that constitutive expression of H3.3K27M at 20°C does not cause fertility defects. The result also shows that the fertility defects depend on elevated temperatures similarly during establishment and maintenance of TEI.

2. Specificity of SET-32 as the H3K23me3 methyltransferase.
The model assigns a central initiating role to SET-32-dependent H3K23me3 deposition. While the genetic requirement for set-32 is convincing, the statement that SET-32 is the only germline H3K23me3 methyltransferase is not supported by current literature.
This conclusion is primarily based on loss of IF signal in set-32 mutants, whereas a recent study (Zhebrun et al., Genetics 2025) identified SET-21 as an additional germline-expressed H3K23 methyltransferase. The authors should revise their wording to reflect SET-32 as a major or dominant enzyme rather than the sole H3K23me3 HMT. Ideally, the authors should test whether SET-21 contributes to initiation or modulates penetrance of the inherited phenotype, or explicitly discuss why SET-21 is unlikely to participate in this paradigm.

We have changed the text in the results section to indicate that SET-32 is the main H3K23me3 HMT at *pachytene* stage. This is consistent with our analysis, and with the finding of Zhebrun et al that SET-21 is mainly expressed during diakinesis oocytes. We now also mention SET-21 as a second H3K23me3 HMT in the discussion, but since Zhebrun et al. report that it contributes less to the H3K23me3 levels, and since the reconstruction of H3K27me3 upon *set-32(RNAi)* is penetrant, we decided to prioritize other experiments over the transgenerational assay upon depletion of SET-21.

3. Relationship between early H3K23me3 and later H3K36me3 domains.
A central claim is that early H3K23me3 deposition enables later accumulation of ectopic H3K36me3, which maintains the inherited state. However, it remains unclear whether the same chromatin regions are involved in this temporal transition.
Specifically:
Are regions initially marked by H3K23me3 the ones that later acquire H3K36me3?
Or are these changes correlated at the nuclear level but not spatially coincident?
Given the reliance on IF-based analyses, additional spatial quantification (e.g. higher-resolution imaging or nucleus-level colocalization metrics) would strengthen the argument for a true chromatin "handoff" rather than parallel global changes.

We agree with the reviewer that it would be desirable to know if the genomic regions occupied by H3K23me3 in early generations are the same as those occupied by H3K36me3 in late generations. However, this would require genomic approaches, which are limited by currently

available protocols for low-input genomic profiling in *C. elegans*, and would therefore require significant method development that are beyond what we could achieve during the revision process.

High resolution imaging is unlikely to answer the question of the handover, as the increased signal of H3K23me3 and the increased signal of H3K36me3 are at least one generation apart. We therefore do not have conditions where we can image the increase of the two histone PTMs together or sequentially in the same animal or nucleus.

To soften our conclusions, we have added a sentence in the discussion that "the prediction from this model that the same genomic regions or nucleosomes that are marked by H3K23me3 during initiation become marked by H3K36me3 remains to be tested".

4. Conceptual relationship between repressive and active-associated histone marks.
H3K23me3 is generally associated with repressive chromatin, whereas H3K36me3 correlates with transcriptionally active gene bodies. How a repressive mark gives rise to, or is replaced by, an active-associated mark remains mechanistically unresolved.
Key questions include:
Is ectopic H3K36me3 driven by transcription in later generations?
Is MES-4 acting in a transcription-independent manner at atypical chromatin domains?
Are transcriptional changes detectable that correlate with the maintenance phase and fertility defects?
While the authors acknowledge the limitations of IF-based profiling, establishing some conceptual or experimental link between chromatin state and transcriptional output would considerably strengthen the mechanistic framework.

This point was also raised by Reviewer 3. To link chromatin state and transcriptional output, have added RNA-seq data from dissected germ lines for P0 and F3 generations. The experiments are explained in more detail in response to Reviewer 3, and the results are shown in new Figure 3C-F and new Figure S2.

In short, we found that the induced chromatin changes in the P0 lead to the ectopic expression of ~1500 mainly somatic genes in the germline of P0array worms, and that largely the same set of genes is still ectopically expressed in subfertile individuals of the F3subfertile generation. The results confirm that the chromatin-based inheritance maintains the same transcriptional output during induction and inheritance.

5. Role of kgb-1 in the inherited phenotype.
The authors show that suppression of kgb-1 partially rescues the P0 fertility phenotype,

implicating stress-response pathways in the initiation phase. However, it remains unclear whether kgb-1 also plays a role in the inherited phenotype.
Does kgb-1 contribute to subfertility or chromatin defects in inheriting generations, or is its role restricted to the initiating P0 context? Testing kgb-1 suppression in F1/F2 inheriting lineages would clarify whether the same effector pathways operate during initiation and maintenance.

We have tested if depleting KGB-1 by RNAi at generations P0-F1, F1-F2, F3-F4 and F4-F5 can suppress the fertility defects. Depleting KGB-1 by RNAi at any generation suppressed the fertility defects in the following generation, as shown in new Figure 3A.

These results indicate that the same effector pathways operate during initiation and maintenance.

Minor points
- Reporting IF variability and nucleus-to-nucleus heterogeneity more explicitly would help interpret penetrance.
The IF heterogeneity per gonad is quantified in Figure 2E. To emphasize on the nucleus-to-nucleus heterogeneity, we have moved what was previously Figure S1B into the main figures (now Figure 2F), as requested by Reviewer 2, point 1.
- Some statements in the Discussion could be phrased more cautiously, given the absence of genome-wide chromatin or transcriptional profiling.
We have changed the discussion as described in response to point 3.

Overall recommendation
This is a conceptually interesting and carefully executed study that would be suitable for EMBO Journal provided the authors address the mechanistic and generality issues outlined above. The work has the potential to make a significant contribution to our understanding of multistep chromatin-based inheritance, but several key conclusions would benefit from clarification and additional support.

Referee #2:

The submission by Ozdemir et al. investigated whether an altered distribution of the critical histone mark H3K27me3 generated in the parental generation is transmitted to offspring. They used C. elegans as their model system and achieved alteration of the distribution of H3K27me3 by expressing H3.3K27M in the parental generation. Previous studies had shown that expression

of H3.3K27M 1) causes H3K27me3 to become less concentrated on the autosomes while it persists on the X chromosomes in the germline and 2) results in endomitotic oocytes and reduced fertility. The authors used a tetracycline-inducible transgene to express H3.3K27M in the parental generation and dialed the tetracycline level to allow worms to have adequate fertility to analyze their offspring in subsequent generations. The transgene also expressed a fluorescent marker, to allow the authors to select offspring that had lost the transgene, so that they could follow the inheritance of altered H3K27me3 without ongoing expression of H3K27M.

The authors analyzed epigenetic inheritance of an altered pattern of H3K27me3 in offspring 1) at the single-cell level by using immunofluorescence to image in individual cells whether the distribution of H3K27me3 remained altered or reverted to wild type and 2) at the whole-organism level by measuring brood size. Based on those two levels of analysis, the major finding is that an altered pattern of H3K27me3 induced in the parental generation and associated reduced fertility are passed to a significant percentage of the offspring for at least 15 generations. Not surprisingly, there is cell-to-cell and organism-to-organism variation. The results support epigenetic transmission of an altered H3K27me3 distribution to some offspring and restoration of a close-to-wild-type H3K27me3 distribution in other offspring.

To investigate initiation and maintenance of an altered H3K27me3 distribution, the authors focused on H3K23me and H3K36me. Both marks displayed more widespread distributions in samples with reduced H3K27me3, and RNAi knock-down of the enzymes that generate each mark influenced the distribution of H3K27me3 and fertility. Based on the generations in which those effects were seen, the authors conclude that H3K23me is important for the initiation phase, and H3K36me is important for the maintenance phase.

All in all, this study uses a clever approach to document the transmission of altered histone marking in one generation to future generations, and to identify key regulators of that transmission.

One limitation of the study (acknowledged by the authors) is the absence of high-resolution information (e.g. from ChIP-seq) on the genome-wide distribution of H3K27me3. But most high-resolution approaches require populations of cells/nuclei/animals and therefore present an average, which would not capture the cell-to-cell variability seen by immunofluorescence and the worm-to-worm variability seen by fertility tests.
We thank the reviewer for the thoughtful comments and suggestions.

Comments:

1) The quality of the documentation of altered distributions of H3K27me3 is variable. The images in Figure 1D are too dark and fuzzy to discern the different staining patterns. Figure 2D is better. Supplemental Figure S1B is good and furthermore illustrates the range of altered patterns and points out in the legend that only complete replicative EI was considered as "altered", whereas full and partial reconstructive EI was considered as "wildtype", as mentioned in lines 223-225 and 393-395. I think that Figure S1B should be moved into the main paper. Additionally, the authors should consider showing that staining in black and white in addition to the merged colors, as red and blue are hard to tease apart in merged images.
We have replaced the fuzzy images in Figure 1D and have moved Figure S1B to the main Figures, now Figure 2F. We have decided against showing the stainings also in black and white, as it made the figure very crowded.

2) The Tet strategy needs a bit more explanation. According to the Mao et al. 2019 reference, it looks like ACTIVATOR is reverse Tet REPRESSOR fused to VP16. Tetracycline exposure causes the ACTIVATOR to bind the TRE - why doesn't the REPRESSOR still bind as well?

To our understanding, the system developed by Mao et al. uses an rtTA activator (binds TRE in presence of Tet) and a tTS repressor (binds TRE in absence of Tet), as is the case in most Tet-Off/Tet-On systems. However, Mao et al. adapted the system to make it effective in *C. elegans* by fusing the rTetR with the QFAD activation domain from the fungal transcription factor QF, and the TetR with the repressor domain from *C. elegans* PIE-1. We have added more details to the text, Figure 1B and the corresponding legend. In any case the system works as advertised, and we only see H3.3K27M expression and the associated phenotypes upon exposure to tetracycline.

3) Lines 167-168 and Figures 1C and 2A differ in whether both P-1 and P0 express H3.3K27M, or only P0. Please clarify.

We have altered Figure 1C to show that in our experimental setup, generations P-1 and P0 were exposed to tetracycline and express K27M.

4) I was confused about generations in Figure 2C and 2E and the associated text in Results. Lines 217-218 say "We determined whether individual P0wt/Fwt or P0array/Fsubfertile animals were fertile or subfertile", which doesn't make sense. In Figure 2E, does the label Fsubfertile refer to the parent of the data points, since the data points show both fertile (triangles) and subfertile (dots). Same question for Figure 2C. Please clarify in the figure and text.

We have changed the wording to clarify the confusion. In the experiments described on lines 217-218, we estimated whether individual animals are fertile or subfertile by letting them lay embryos until the second day of adulthood, and then carried out IF of these same animals. This approach allows us to conclude that subfertile animals have an increased number of germ cells with altered H3K27me3 landscapes, while fertile animals in the same generation have a very low number of germ cells with altered H3K27me3 landscapes (Figure 2E).

The data points in both Figures 2C and 2E indeed refer to the animals in the generation indicated on the x-axis label (brood size of animal in P0, F1 etc in panel C, and % pachytene nuclei per gonad of animals in P0, F1 etc). We will more explicitly state this in the figure legend.

Since the penetrance of inheritance of subfertility is about 30%, some animals in the F1subfertile generation will have broodsizes above 50. These appear as datapoints in the F1 generation above the dashed line in Figure 2C. When we carried out IF analysis, we found that these animals of the F1subfertile generation with broodsizes above 50 had a low percentage of nuclei with altered H3K27me3 landscapes. This is reflected by the triangles in the F1 generation of Figure 2E. The same applies for the F2subfertile generation etc. We have made textual changes to make this more clear.

5) In Figure 4C, should the generations at the bottom be P0, F1, F2 instead of F1, F2, F3?

Yes, the reviewer is correct! We have corrected this mistake.

Line 188 - fix "sub-phenotyes"
Line 206 - should Figure 1D be cited, instead of Figure 1C?
Line 531 - delete "chromodomain proteins"

We have changed the wording on line 188 to avoid having “sub-phenotype” and “subfertile” in the same sentence. The sentence now reads “The offspring of subfertile worms displayed both *subfertile* and *fertile* phenotypes in each generation, implying that individual worms can both replicate the *H3.3K27M*^{tet}-induced changes or reconstruct the wildtype H3K27me3 landscape, respectively, in different germ cells.”

We have corrected the other mistakes.

Referee #3:

In this manuscript, the authors investigate transgenerational epigenetic inheritance of chromatin defects due to the effect of transient expression of an H3.3K27M. This has been

shown before to lead to genome-wide chromatin changes in C. elegans but what the authors do now is to use this system to study the inheritance of the disordered H3K27me3 that results from H3.3K27M overexpression over several generations. This is because they can link phenotype (brood size) to H3K27me3 levels (via IF) at the single individual level, essential as the inheritance of the disordered chromatin is somewhat stochastic.
Overall, within the framework of the experiments performed the results are solid, with appropriate statistics and controls, and convincing. The weakness, however, is that the authors cannot provide a clear molecular link between the fertility defects and the 'disordered' landscape because IF is such a blunt tool, which cannot identify the specific genes, or set of genes, that are responsible. This is of fundamental importance because the model that the authors propose is that there is stochastic, imperfect, inheritance of the defective landscape- this implies, though, that even individual worms with disordered H3K27me3 as measured by IF, might actually differ in which regions of the genome are affected. It's obviously very hard to deal with this variability at the chromatin level, because a genome-wide ChIP-seq is impractical from single worms. However, there are some approaches that could be taken:
i) could pools of worms assayed for low fertility be used for ChIP-seq experiments? For example, many single worms could be plated, allowed to lay eggs for a fixed period, and then frozen in an individual tube. After analysing how many eggs hatched (brood size) the worms in the different tubes could be pooled into either high or low fertility groups and ChIP done on them. I think that 100 worms for each group (so possibly 200 total) would be enough. This would be a considerable effort, but I think would be possible.
ii) Single worm RNAseq has been developed (see for example https://www.biorxiv.org/content/10.1101/2025.11.01.686022v1 or https://elifesciences.org/articles/106525). This could be combined with fertility assay (as above) and each worm studied for its RNA profile. The key question would be whether there are predictable gene expression correlates associated with fertility defects, ie that most worms showing the H3K27M induced defects will have changes in expression of certain genes. These could be linked to chromatin domains according to known maps of WT chromatin, to see whether (say) upregulated genes are enriched for H3K27M domains.
I feel that, for a journal such as EMBO, something to bolster the molecular chain of causation between defective H3K27me3 and fertility and transgenerational epigenetic inheritance would be important, so perhaps the authors could think about these suggestions, or alternatively think of something else that might address this key point.

We thank the reviewer for the thoughtful comments and suggestions. We agree with the reviewer both on the desirability of higher resolution maps, and the experimental challenges that we face to get them. We appreciate the suggested experiments and references. ChIP-seq for very low input samples is not something that we could develop in the scope of this revision.

Moreover, we are skeptical that ChIP-seq experiments for determining H3K27me3 patterns would allow much further insight. We would still average the variable patterns within the germline of multiple individuals. We would likely come to the same conclusion as from the IF experiments – loss on most of the autosomes, and retention on the X chromosome and some autosomal domains.

We have carried out single worm RNA-seq using the protocol in the publications referenced by the reviewer. The results were technically variable, with many samples yielding little or no RNA. For the few worms that we could profile, the different groups (subfertile individuals from P0array, fertile and subfertile individuals from F3subfertile) clustered together and showed only a very small number of differentially expressed genes. This makes no biological sense and is likely due to the sparsity of the reads and the high variability between individual worms within the same samples.

We therefore dissected germlines of adults from subfertile individuals from P0array, fertile individuals from P0wt, and fertile or subfertile individuals from F3subfertile. We sequenced pools of 20 germlines each. The results are shown in new Figure 3C-F and new Figure S2. They show that the induced changes of the H3K27me3 landscape lead to the ectopic expression of ~1500 mainly somatic genes in the germline of P0array worms. Largely the same set of genes is still ectopically expressed in subfertile worms of the F3subfertile generation (overlap ~1000 genes), confirming that the chromatin-based inheritance maintains the same downstream effects during induction and inheritance. Worms that have recovered their fertility to wildtype levels among the F3subfertile showed gene expression patterns that are very similar to wildtype worms, further demonstrating that changed H3K27me3, aberrant gene expression and fertility defects are causally linked.

The RNA-seq analysis of the dissected germlines integrates the chromatin and fertility defects, and shows a molecular chain of causation between defective H3K27me3, aberrant gene expression and fertility during transgenerational epigenetic inheritance.

Prof. Florian Steiner
University of Geneva
Department of Molecular and Cellular Biology
Switzerland

3rd Aug 2026

Re: EMBOJ-2025-123096R
Transgenerational inheritance of altered H3K27me3 in wildtype Caenorhabditis elegans

Dear Florian,

Thank you for submitting your revised manuscript on transgenerational H3K27 methylation inheritance for our consideration. Two of the original referees have now reviewed it once more, and overall found the study significantly strengthened and now suitable for publication, pending a few interpretational/textual modifications still requested by referee 1. In addition, there are also various editorial points to be incorporated at this stage:

- Please adjust the order of the manuscript sections, and also make sure to use the correct section headers:
Single title page with complete author information and a mark indicating the corresponding author(s) in the list, Abstract, Introduction, Results, Discussion, Methods, Data Availability, Acknowledgements, Disclosure and Competing Interests Statement, References, Main Figure Legends, Tables, Expanded Figure Legends.

- As we are switching from a free-text author contribution statement towards a more formal statement based on Contributor Role Taxonomy (CRediT) terms, please remove the present Author Contribution section and instead specify each author's contribution(s) directly in the Author Information page of our submission system during upload of the final manuscript. See https://casrai.org/credit/ for more information.

- Please adjust the format of the reference list according to EMBO Journal format. In particular, make sure that all references have their complete citation information - including year, journal name & volume, and page/elocator numbers. On the other hand, please mention DOI information/URLs only in case of advance publications that do not (yet) have volume/page numbers. Finally, for references with multiple authors, only the first up to 10 authors should be listed, followed by 'et al.' after that.

- As we have switched from a free-text author contribution statement towards a more formal statement based on Contributor Role Taxonomy (CRediT) terms, please remove the present Author Contribution section and instead specify each author's contribution(s) directly in the Author Information page of our submission system during upload of the final manuscript. See https://casrai.org/credit/ for more information.

- Please check that each figure panel is referenced at least once in the text (this currently appears to be missing for the EV Figures) - in case you want to call out a whole figure in completeness, please simply use e.g. 'Figure EV3A-C'.

- In the Source Data checklist, please use the comments boxes at the bottom to point readers to any YARETA-deposited data for a given figure/panel. For the numerical source data that currently appears to be included in 'Table EV1', we would ask to upload these raw data instead as Source Data files -one XLSX or ZIP file per each main figure- and to remove all references to Table EV1 from the text.

- Please provide a simple synopsis image, which can be used as a "visual title" for the synopsis section of your paper (maybe based on Fig. 6?). The image should be in JPG format of 550 pixels wide and 300 pixels high, and please make sure that it remains clear enough even in these modest dimensions.

- Finally, during routine pre-acceptance checks, our data editors have raised the following queries regarding figures, data, and legends; I would appreciate if you briefly answered to them in the cover letter of your final submission, and made the requested text modifications with changes/additions highlighted via the "Track changes" option, to facilitate our final checking.
1. Please provide information related to 'n' in the legends of figures 1E,F,G.
2. Please define the box plots in terms of centre, minima, maxima, and whiskers, bounds of box and percentile in the legends of figure 1E.
3. Please provide the exact p values in the legends of figures 1F,G; 2E; 3A,B; 4B; 5B,D.
4. Please indicate the statistical test used for data analysis in the legends of figure 3C.
5. Please define the scale bar for figure EV-3B.

I am returning the manuscript to you for a final round of minor revision, to allow you to make the remaining adjustments and re-upload all modified files using the link below. Once we will have received them, we should be ready to swiftly proceed with

formal acceptance and production of the manuscript.

With kind regards,

Hartmut

Hartmut Vodermaier, PhD
Senior Editor, The EMBO Journal
h.vodermaier@embojournal.org

*** PLEASE NOTE: All revised manuscript are subject to initial checks for completeness and adherence to our formatting guidelines. Revisions may be returned to the authors and delayed in their editorial re-evaluation if they fail to comply to the following requirements. As a first step please read our guidelines for revised submissions:
https://link.springer.com/journal/44318/submission-guidelines#cms-Revised-submissions

1) Every manuscript requires a Data Availability section (even if only stating that no deposited datasets are included). Primary datasets or computer code produced in the current study have to be deposited in appropriate public repositories prior to resubmission, and reviewer access details provided in case that public access is not yet allowed.

2) Each figure legend must specify
- size of the scale bars that are mandatory for all micrograph panels
- the statistical test used to generate error bars and P-values
- the type error bars (e.g., S.E.M., S.D.)
- the number (n) and nature (biological or technical replicate) of independent experiments underlying each data point
- Figures may not include error bars for experiments with n<3; scatter plots showing individual data points should be used instead.

3) Revised manuscript text (including main tables, and figure legends for main and EV figures) has to be submitted as editable text file (e.g., .docx format). We encourage highlighting of changes (e.g., via text color) for the referees' reference.

4) Each main and each Expanded View (EV) figure should be uploaded as individual production-quality files (preferably in .eps, .tif, .jpg formats). For suggestions on figure preparation/layout, please refer to our Figure Preparation Guidelines:
https://media.springernature.com/original/springer-cms/rest/v1/content/27825798/data/v1

5) Point-by-point response letters should include the original referee comments in full together with your detailed responses to them (and to specific editor requests if applicable), and also be uploaded as editable (e.g., .docx) text files.

6) Please complete our Author Checklist, and make sure that information entered into the checklist is also reflected in the manuscript; the checklist will be available to readers as part of the Review Process File.

7) All authors listed as (co-)corresponding need to deposit, in their respective author profiles in our submission system, a unique ORCiD identifier linked to their name. Please see our Guide to Authors for detailed instructions.

8) Please note that supplementary information at EMBO Press has been superseded by the 'Expanded View' for inclusion of additional figures, tables, movies or datasets; with up to five EV Figures being typeset and directly accessible in the HTML version of the article.

9) To facilitate reproducibilty and cross-laboratory adoption of methodologies, please structure the Materials & Methods section as outlined in our guide to authors, including a completed Reagents and Tools Table.

10) Digital image enhancement is acceptable practice, as long as it accurately represents the original data and conforms to community standards. If a figure has been subjected to significant electronic manipulation, this must be clearly noted in the figure legend and/or the 'Materials and Methods' section. The editors reserve the right to request original versions of figures and the original images that were used to assemble the figure. Finally, we generally encourage uploading of numerical as well as gel/blot image source data.

At EMBO Press we ask authors to provide source data for the main manuscript figures. You will receive a separate email with instructions for providing source data with your revised manuscript, including how to upload and organize the files.

In the interest of ensuring the conceptual advance provided by the work, we recommend submitting a revision within 3 months (1st Nov 2026). Please discuss the revision progress ahead of this time with the editor if you require more time to complete the

revisions. Use the link below to submit your revision:

Link Not Available

Referee #1:

The authors have made a substantial effort to address my previous concerns. The new temperature-shift and kgb-1 RNAi experiments clarify the temperature dependence and downstream phenotypic consequences of the inherited state, while the germline RNA-seq data provide evidence that related transcriptional alterations persist in inheriting generations. The revised discussion of SET-32 and SET-21 is also appropriate, and the improved presentation of nucleus-to-nucleus heterogeneity strengthens the manuscript. I am therefore broadly supportive of publication.

A few points should nevertheless be addressed before acceptance:
1. The proposed initiation-to-maintenance model is reasonable and provides a useful synthesis of the temporal and genetic data. However, the experiments do not establish that H3K23me3 and H3K36me3 sequentially occupy the same genomic regions or nucleosomes. The authors acknowledge this in the Discussion, but the Results and Figure 6 legend still state too definitively that H3K36me3 "replaces" H3K23me3. This transition should be presented explicitly as a proposed or inferred handoff, and this limitation should also be added to the Limitations section. No additional experiment is essential.
2. The temperature-shift experiments show that growth at 20{degree sign}C suppresses the fertility phenotype, but H3K27me3 patterns were not examined after the shift. The authors should therefore distinguish phenotypic suppression from erasure or reconstruction of the inherited chromatin state.
3. The RNA-seq data provide valuable support for the persistence of a related transcriptional state, but statements such as "molecular chain of causation" or "causally linked" appear stronger than warranted. These should be replaced by more cautious wording reflecting an association between chromatin changes, transcriptional alterations, and fertility.
4. The overlap reported for the RNA-seq data should be checked. Figure 3D appears to indicate 1,126 genes shared between subfertile P0 and F3 animals, whereas the text reports 1,156.
5. The concluding claim that the inherited state represents a mechanism of adaptation appears somewhat stronger than the data support. The study demonstrates the persistence of epigenetic variation under experimental selection, but does not test whether the inherited phenotype confers a fitness advantage in a defined environment. The authors should either qualify the adaptation language, particularly in the Abstract, or clarify that this is a broader conceptual implication rather than a direct conclusion of the study.

Subject to these mainly interpretative and textual revisions, I consider the manuscript suitable for publication.

Referee #3:

I thank the authors for responding to my questions in the first round of review. They have made a good attempt at addressing them, given the technical difficulties in assessing the actual histone modifications themselves, and I think that the manuscript now reaches the standard I'd expect for a paper in EMBOJ.

Response to Reviewers 2

Referee #1:

The authors have made a substantial effort to address my previous concerns. The new temperature-shift and kgb-1 RNAi experiments clarify the temperature dependence and downstream phenotypic consequences of the inherited state, while the germline RNA-seq data provide evidence that related transcriptional alterations persist in inheriting generations. The revised discussion of SET-32 and SET-21 is also appropriate, and the improved presentation of nucleus-to-nucleus heterogeneity strengthens the manuscript. I am therefore broadly supportive of publication.
We thank the reviewer for the kind and constructive feedback.

A few points should nevertheless be addressed before acceptance:
1. The proposed initiation-to-maintenance model is reasonable and provides a useful synthesis of the temporal and genetic data. However, the experiments do not establish that H3K23me3 and H3K36me3 sequentially occupy the same genomic regions or nucleosomes. The authors acknowledge this in the Discussion, but the Results and Figure 6 legend still state too definitively that H3K36me3 "replaces" H3K23me3. This transition should be presented explicitly as a proposed or inferred handoff, and this limitation should also be added to the Limitations section. No additional experiment is essential.
We have added this point to the limitations (which is now the last discussion paragraph). Figure 6 is explicitly a model figure depicting nuclei, not individual genomic regions or nucleosomes. We feel that in this context and at this level, the replacement of H3K23me3 by H3K36me3 matches our observation, and the description is therefore accurate. This is also the case for the last Results paragraph, where we explicitly state that the text describes a model that we propose.
2. The temperature-shift experiments show that growth at 20{degree sign}C suppresses the fertility phenotype, but H3K27me3 patterns were not examined after the shift. The authors should therefore distinguish phenotypic suppression from erasure or reconstruction of the inherited chromatin state.
We have altered the text to "These results demonstrate that the fertility defects can be suppressed by the same treatment in both the $P0^{array}$ and the $F^{subfertile}$, supporting the notion that the underlying cause of the phenotype remains the same during TEI. " This statement avoids making a direct link to the chromatin states.
3. The RNA-seq data provide valuable support for the persistence of a related transcriptional state, but statements such as "molecular chain of causation" or "causally linked" appear stronger than warranted. These should be replaced by more cautious wording reflecting an association between chromatin changes, transcriptional alterations, and fertility.
We have removed the words "causally" and "molecular chain of causation", now stating "demonstrating that changed H3K27me3 patterns, aberrant gene expression and fertility

defects are linked" and "provides a molecular link between H3K27me3 patterns, gene expression and fertility"

4. The overlap reported for the RNA-seq data should be checked. Figure 3D appears to indicate 1,126 genes shared between subfertile P0 and F3 animals, whereas the text reports 1,156.

We have corrected the number to 1126 in the text.

5. The concluding claim that the inherited state represents a mechanism of adaptation appears somewhat stronger than the data support. The study demonstrates the persistence of epigenetic variation under experimental selection, but does not test whether the inherited phenotype confers a fitness advantage in a defined environment. The authors should either qualify the adaptation language, particularly in the Abstract, or clarify that this is a broader conceptual implication rather than a direct conclusion of the study.

We have softened the concluding sentence to "We propose that this inheritance of epigenome-based phenotypes can provide a mutation-independent mechanism for adaptation to the environment.", and replaced "demonstrate" with "indicate" in the abstract.

Subject to these mainly interpretative and textual revisions, I consider the manuscript suitable for publication.

Referee #3:

I thank the authors for responding to my questions in the first round of review. They have made a good attempt at addressing them, given the technical difficulties in assessing the actual histone modifications themselves, and I think that the manuscript now reaches the standard I'd expect for a paper in EMBOJ.

We thank the reviewer for the kind and constructive feedback.

EMBO Press Author Checklist

Corresponding Author Name: Florian A. Steiner
Journal Submitted to: The EMBO Journal
Manuscript Number: EMBOJ-2025-123096R

USEFUL LINKS FOR COMPLETING THIS FORM
The EMBO Journal - Author Guidelines
EMBO Reports - Author Guidelines
Molecular Systems Biology - Author Guidelines
EMBO Molecular Medicine - Author Guidelines

Reporting Checklist for Life Science Articles (updated January
This checklist is adapted from Materials Design Analysis Reporting (MDAR) Checklist for Authors. MDAR establishes a minimum set of requirements in transparent reporting in the life sciences (see Statement of Task: 10.31222/osf.io/9sm4x). Please follow the journal's guidelines in preparing your
Please note that a copy of this checklist will be published alongside your article.

Abridged guidelines for figures
1. Data
The data shown in figures should satisfy the following conditions:
- the data were obtained and processed according to the field's best practice and are presented to reflect the results of the experiments in an accurate and unbiased manner.
- ideally, figure panels should include only measurements that are directly comparable to each other and obtained with the same assay.
- plots include clearly labeled error bars for independent experiments and sample sizes. Unless justified, error bars should not be shown for technical
- if n<5, the individual data points from each experiment should be plotted. Any statistical test employed should be justified.
- Source Data should be included to report the data underlying figures according to the guidelines set out in the authorship guidelines on Data

2. Captions
Each figure caption should contain the following information, for each panel where they are relevant:
- a specification of the experimental system investigated (eg cell line, species name).
- the assay(s) and method(s) used to carry out the reported observations and measurements.
- an explicit mention of the biological and chemical entity(ies) that are being measured.
- an explicit mention of the biological and chemical entity(ies) that are altered/varied/perturbed in a controlled manner.
- the exact sample size (n) for each experimental group/condition, given as a number, not a range;
- a description of the sample collection allowing the reader to understand whether the samples represent technical or biological replicates (including how many animals, litters, cultures, etc.).
- a statement of how many times the experiment shown was independently replicated in the laboratory.
- definitions of statistical methods and measures:
  - common tests, such as t-test (please specify whether paired vs. unpaired), simple χ2 tests, Wilcoxon and Mann-Whitney tests, can be unambiguously identified by name only, but more complex techniques should be described in the methods section;
  - are tests one-sided or two-sided?
  - are there adjustments for multiple comparisons?
  - exact statistical test results, e.g., P values = x but not P values < x;
  - definition of 'center values' as median or average;
  - definition of error bars as s.d. or s.e.m.

Please complete ALL of the questions below.
Select "Not Applicable" only when the requested information is not relevant for your study.

Materials

Newly Created Materials	Information included in the manuscript?	In which section is the information available? (Reagents and Tools Table, Materials and Methods, Figures, Data Availability Section)
New materials and reagents need to be available; do any restrictions apply?	Yes	Materials and Methods

Antibodies	Information included in the manuscript?	In which section is the information available? (Reagents and Tools Table, Materials and Methods, Figures, Data Availability Section)
For **antibodies** provide the following information:<br>- Commercial antibodies: RRID (if possible) or supplier name, catalogue number and or/clone number<br>- Non-commercial: RRID or citation	Yes	Reagents and Tools table

DNA and RNA sequences	Information included in the manuscript?	In which section is the information available? (Reagents and Tools Table, Materials and Methods, Figures, Data Availability Section)
**Short novel DNA or RNA including primers, probes**: provide the sequences.	Not Applicable	

Cell materials	Information included in the manuscript?	In which section is the information available? (Reagents and Tools Table, Materials and Methods, Figures, Data Availability Section)
**Cell lines:** Provide species information, strain. Provide accession number in repository **OR** supplier name, catalog number, clone number, and/**OR** RRID.	Not Applicable	
**Primary cultures:** Provide species, strain, sex of origin, genetic modification status.	Not Applicable	
Report if the cell lines were recently **authenticated** (e.g., by STR profiling) and tested for mycoplasma contamination.	Not Applicable	

Experimental animals	Information included in the manuscript?	In which section is the information available? (Reagents and Tools Table, Materials and Methods, Figures, Data Availability Section)
**Laboratory animals or Model organisms:** Provide species, strain, sex, age, genetic modification status. Provide accession number in repository **OR** supplier name, catalog number, clone number, **OR** RRID.	Yes	Materials and Methods
**Animal observed in or captured from the field:** Provide species, sex, and age where possible.	Not Applicable	
Please detail **housing and husbandry conditions**.	Not Applicable	

Plants and microbes	Information included in the manuscript?	In which section is the information available? (Reagents and Tools Table, Materials and Methods, Figures, Data Availability Section)
**Plants:** provide species and strain, ecotype and cultivar where relevant, unique accession number if available, and source (including location for collected wild specimens).	Not Applicable	
**Microbes:** provide species and strain, unique accession number if available, and source.	Not Applicable	

Human research participants	Information included in the manuscript?	In which section is the information available? (Reagents and Tools Table, Materials and Methods, Figures, Data Availability Section)
If collected and within the bounds of privacy constraints report on age, sex and gender or ethnicity for all study participants.	Not Applicable	

Core facilities	Information included in the manuscript?	In which section is the information available? (Reagents and Tools Table, Materials and Methods, Figures, Data Availability Section)
If your work benefited from core facilities, was their service mentioned in the acknowledgments section?	Yes	Acknowledgments

Design

**Study protocol**	**Information included in the manuscript?**	**In which section is the information available?** (Reagents and Tools Table, Materials and Methods, Figures, Data Availability Section)
If study protocol has been **pre-registered, provide DOI in the manuscript**. For clinical trials, provide the trial registration number **OR** cite DOI.	Not Applicable	
Report the **clinical trial registration number** (at ClinicalTrials.gov or equivalent), where applicable.	Not Applicable	

**Laboratory protocol**	**Information included in the manuscript?**	**In which section is the information available?** (Reagents and Tools Table, Materials and Methods, Figures, Data Availability Section)
Provide DOI OR other citation details if **external detailed step-by-step protocols** are available.	Not Applicable	

**Experimental study design and statistics**	**Information included in the manuscript?**	**In which section is the information available?** (Reagents and Tools Table, Materials and Methods, Figures, Data Availability Section)
Include a statement about **sample size** estimate even if no statistical methods were used.	Yes	Figures
Were any steps taken to minimize the effects of subjective bias when allocating animals/samples to treatment (e.g. **randomization procedure**)? If yes, have they been described?	Yes	Materials and Methods
Include a statement about **blinding** even if no blinding was done.	Not Applicable	
Describe **inclusion/exclusion criteria** if samples or animals were excluded from the analysis. Were the criteria pre-established?<br><br>If sample or data points were omitted from analysis, report if this was due to attrition or intentional exclusion and provide justification.	Not Applicable	
For every figure, are **statistical tests** justified as appropriate? Do the data meet the assumptions of the tests (e.g., normal distribution)? Describe any methods used to assess it. Is there an estimate of variation within each group of data? Is the variance similar between the groups that are being statistically compared?	Yes	Figures

**Sample definition and in-laboratory replication**	**Information included in the manuscript?**	**In which section is the information available?** (Reagents and Tools Table, Materials and Methods, Figures, Data Availability Section)
In the figure legends: state number of times the experiment was **replicated** in laboratory.	Yes	Figures
In the figure legends: define whether data describe **technical or biological replicates**.	Yes	Materials and Methods

Ethics

**Ethics**	**Information included in the manuscript?**	**In which section is the information available?** (Reagents and Tools Table, Materials and Methods, Figures, Data Availability Section)
Studies involving **human participants**: State details of **authority granting ethics approval** (IRB or equivalent committee(s), provide reference number for approval.	Not Applicable	
Studies involving **human participants**: Include a statement confirming that **informed consent** was obtained from all subjects and that the experiments conformed to the principles set out in the WMA Declaration of Helsinki and the Department of Health and Human Services Belmont Report.	Not Applicable	
Studies involving **human participants:** For publication of **patient photos**, include a statement confirming that consent to publish was obtained.	Not Applicable	
Studies involving experimental **animals**: State details of **authority granting ethics approval** (IRB or equivalent committee(s), provide reference number for approval. Include a statement of compliance with ethical regulations.	Not Applicable	
Studies involving **specimen and field samples:** State if relevant **permits** obtained, provide details of authority approving study; if none were required, explain why.	Not Applicable	

**Dual Use Research of Concern (DURC)**	**Information included in the manuscript?**	**In which section is the information available?** (Reagents and Tools Table, Materials and Methods, Figures, Data Availability Section)
Could your study fall under dual use research restrictions? Please check biosecurity documents and list of **select agents and toxins** (CDC): https://www.selectagents.gov/sat/list.htm	Not Applicable	
If you used a select agent, is the security level of the lab appropriate and reported in the manuscript?	Not Applicable	
If a study is subject to dual use research of concern regulations, is the name of the **authority granting approval and reference number** for the regulatory approval provided in the manuscript?	Not Applicable	

Reporting

The MDAR framework recommends adoption of discipline-specific guidelines, established and endorsed through community initiatives. Journals have their own policy about requiring specific guidelines and recommendations to complement MDAR.

**Adherence to community standards**	**Information included in the manuscript?**	**In which section is the information available?** (Reagents and Tools Table, Materials and Methods, Figures, Data Availability Section)
State if relevant guidelines or checklists (e.g., **ICMJE, MIBBI, ARRIVE, PRISMA**) have been followed or provided.	Not Applicable	
For **tumor marker prognostic studies,** we recommend that you follow the **REMARK** reporting guidelines (see link list at top right). See author guidelines, under 'Reporting Guidelines'. Please confirm you have followed these guidelines.	Not Applicable	
For **phase II and III randomized controlled trials**, please refer to the **CONSORT** flow diagram (see link list at top right) and submit the CONSORT checklist (see link list at top right) with your submission. See author guidelines, under 'Reporting Guidelines'. Please confirm you have submitted this list.	Not Applicable	

Data Availability

**Data availability**	**Information included in the manuscript?**	**In which section is the information available?** (Reagents and Tools Table, Materials and Methods, Figures, Data Availability Section)
Have **primary datasets** been deposited according to the journal's guidelines (see 'Data Deposition' section) and the respective accession numbers provided in the Data Availability Section?	Yes	Data availability
Were **human clinical and genomic datasets** deposited in a public access-controlled repository in accordance to ethical obligations to the patients and to the applicable consent agreement?	Not Applicable	
Are **computational models** that are central and integral to a study available without restrictions in a machine-readable form? Were the relevant accession numbers or links provided?	Not Applicable	
If publicly available data were reused, provide the respective **data citations in the reference list**.	Not Applicable	
