# Supplementary material for: Transgenerational inheritance of altered H3K27me3 in wild-type Caenorhabditis elegans
Source: EMBO J. 2026 Sep 18;45(20):7226–45. doi: 10.1038/s44318-026-00907-9 (PMC13672289; doi:10.1038/s44318-026-00907-9)
Supplement: Supplementary file 2 [file 44318_2026_907_MOESM2_ESM.pdf]

Expanded View Figures

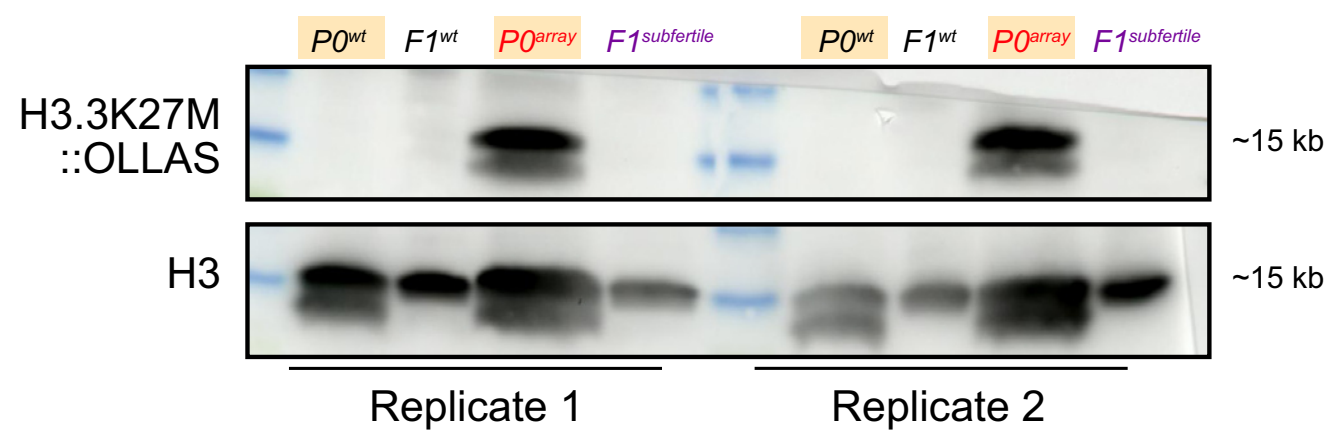

Figure EV1. *H3.3K27M^{tet}* expression is restricted to P0.

Detection of H3.3K27M protein by western blots in tetracycline-exposed *P0*^{array} and *F*^{subfertile}, with *P0*^{wt} and *F1*^{wt} worms as controls. The OLLAS tag was used to detect H3.3K27M, and H3 was used as a loading control. Whole-worm lysates of 40 adult worms were loaded in each lane. A beige background signifies that the worms were grown on media containing 1 ng/µl tetracycline.

 © The Author(s)

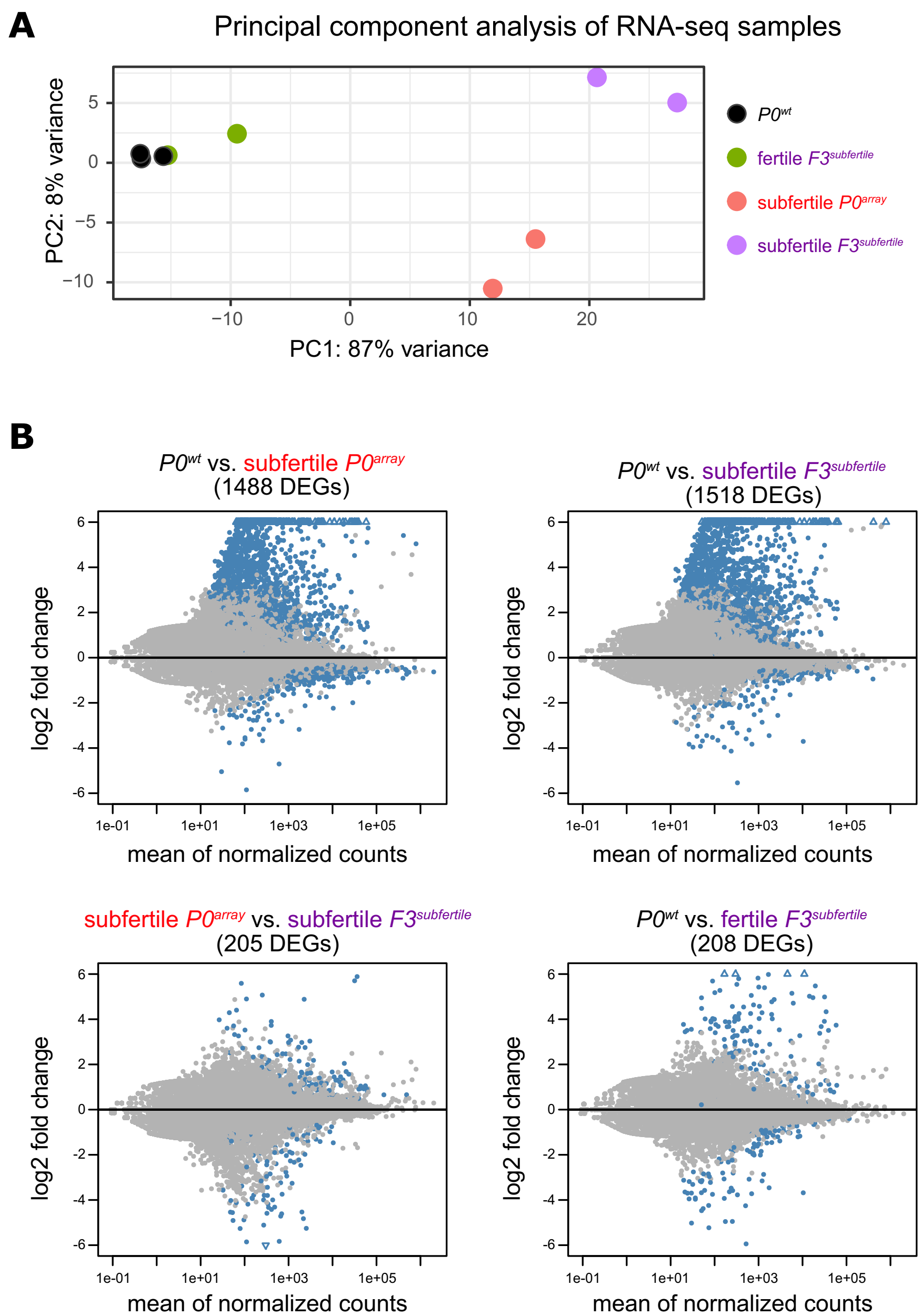

Figure EV2. RNA-seq analysis of germ lines in the parental and inheriting generations.

Germ lines were dissected from *P0*^{wt} (wildtype control; $n = 3$), subfertile individuals from *P0*^{array} ($n = 2$), subfertile individuals from *F3*^{subfertile} ($n = 2$), and fertile individuals from *F3*^{subfertile} ($n = 2$). Each sample contained a pool of about 20 dissected germ lines. (**A**) PCA analysis of the RNA-seq samples, showing that biological replicates cluster together, and samples from fertile individuals are distinct from samples from subfertile individuals. (**B**) Volcano plots for pairwise comparison of the indicated samples. Differentially expressed genes (DEGs) were identified based on a fold change of ≥2 and an FDR of ≤0.01 and are shown in blue.

© The Author(s)

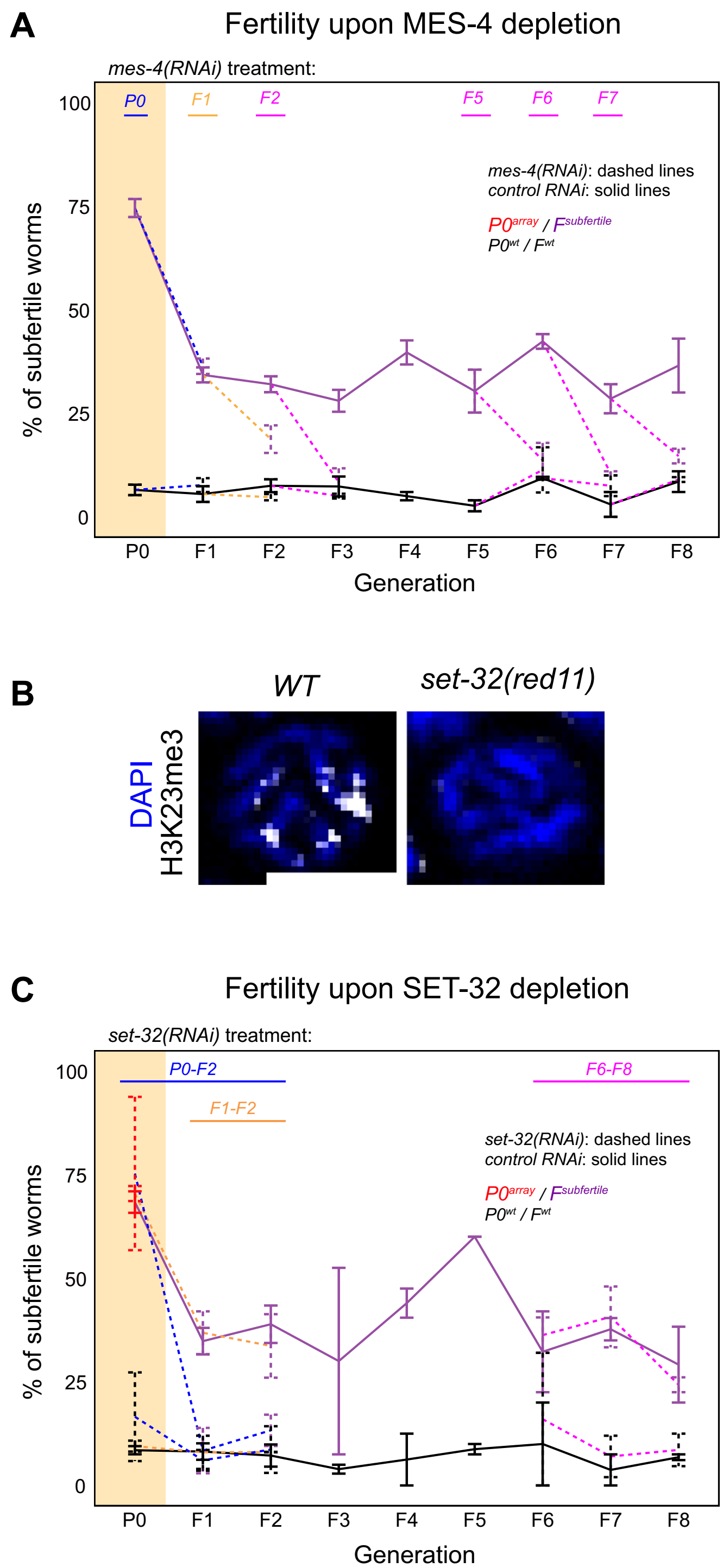

Figure EV3. Transgenerational infertility assay upon HMT depletion.

(**A**) Transgenerational infertility assay upon *mes-4(RNAi)* treatment. Mean percentages of subfertile worms in generations $P0^{wt}$-$F8^{wt}$ (black, and dashed lines) and $P0^{array}$-$F8^{subfertile}$ (purple, and dashed lines). Animals were exposed to *mes-4(RNAi)* treatment (dashed lines) in P0 (blue), F1 (orange), F2, F5, F6, or F7 (fuchsia), and the fertility of the following generation was assessed. Animals were exposed to control RNAi treatment (solid lines) in parallel. Means with standard errors of at least two independent transgenerational assays are shown. (**B**) SET-32 is the main writer for H3K23me3 in pachytene nuclei. Representative images from immunostaining of H3K23me3 (white) in pachytene nuclei of wild-type and *set-32(red11)* animals grown at 25 °C. Scale bar represents 4 μm. (**C**) Transgenerational infertility assay upon *set-32(RNAi)* treatment. Mean percentages of subfertile worms in generations $P0^{wt}$-$F8^{wt}$ (black and dashed lines) and $P0^{array}$-$F8^{subfertile}$ (purple and dashed lines). Animals were exposed to *set-32(RNAi)* treatment (dashed lines) from P0 to F2 (blue), or from F1 to F2 (orange), or from F6 to F8 (fuchsia). Animals were exposed to control RNAi treatment (solid lines) in parallel. Means and standard errors of at least three independent transgenerational assays are shown.

 © The Author(s)
